# Chloroplast Genome Sequencing and Comparative Analysis of Six Medicinal Plants of *Polygonatum*

**DOI:** 10.1002/ece3.70831

**Published:** 2025-01-10

**Authors:** Jinchen Yao, Zhaohuan Zheng, Tao Xu, Duomei Wang, Jingzhe Pu, Yazhong Zhang, Liangping Zha

**Affiliations:** ^1^ College of Pharmacy Anhui University of Chinese Medicine Hefei China; ^2^ Biological and Pharmaceutical Engineering West Anhui University Luan China; ^3^ Anhui Institute for Food and Drug Control Hefei China; ^4^ Institute of Conservation and Development of Traditional Chinese Medicine Resources Anhui Academy of Chinese Medicine Hefei China; ^5^ MOE‐Anhui Joint Collaborative Innovation Center for Quality Improvement of Anhui Genuine Chinese Medicinal Materials Hefei China; ^6^ Joint Research Center for Chinese Herbal Medicine of Anhui of IHM, Anhui University of Chinese Medicine Hefei China

**Keywords:** chloroplast genome, interspecific relationships, medicinal plants, phylogenetic, *Polygonatum*

## Abstract

The genus *Polygonatum* boasts abundant germplasm resources and comprises numerous species. Among these, medicinal plants of this genus, which have a long history, have garnered attention of scholars. This study sequenced and analyzed the chloroplast genomes of six species of *Polygonatum* medicinal plants (*P. zanlanscianense*, *P. kingianum*, *P. sibiricum* , *P. cyrtonema*, *P. filipes* , and *P. odoratum*, respectively) to explore their interspecific relationships. The sequence length (154, 578–155, 807 bp) and genome structure were conserved among the six *Polygonatum* species, with a typical tetrad structure. Among the 127–131 genes contained in the genomes, 84–85 are protein‐coding genes, 37–38 are transfer RNA genes, and 6–8 are ribosomal RNA genes. The genomes contained 64–76 simple sequence repeats (SSRs) and 36–62 long repetitive sequences. Codon bias patterns tended to use codons ending in A/T. In 30 types of codons with RSCU > 1, 93.3% ended in A/T of the six species. Twenty‐one highly variable plastid regions were identified in the chloroplast genomes of the six medicinal plants. Furthermore, a phylogenetic analysis encompassing these and 53 other chloroplast genomes of *Polygonatum* species revealed that *P. cyrtonema*, *P. odoratum*, and *P. filipes* clustered together on one clade, whereas *P. kingianum* and *P. zanlanscianense* formed separate clades. Notably, *P. sibiricum* emerged as a standalone clade, and our phylogenetic tree reinforces the classification of *P. sibiricum* as forming a monophyly. This study provides a novel basis for intragenus taxonomy and DNA barcoding molecular identification within the genus *Polygonatum* medicinal plants.

## Graphical abstract

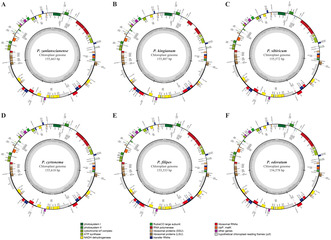

## 1 Introduction

*Polygonatum* Mill. is the largest genus in the Polygonateae tribe. The genus comprises approximately 70 species (Gong et al. 2023), of which 37 species and 1 variety have records of medicinal use. *Polygonatum* spp., as well as Huangjing, has been used medicinally for more than 2000 years in China. It was first recorded in Hongjing Tao's *Mingyi Bie Lu* and was considered as “top‐grade” Chinese medicine. Pharmacological studies show that *Polygonatum* spp. have biological activities on anti‐inflammatory, anticancer, antioxidant, anti‐aging, and hypoglycemic (Zhao et al. 2018, 2020; Luo et al. 2022; Yang et al. 2023). Among this genus, *Polygonatum kingianum*, *P. sibiricum* , *P. cyrtonema*, and *P. odoratum* are collected in the Chinese Pharmacopeia as the official source of Polygonati rhizoma and Polygonate odorati rhizome. *P. filipes* and *P. zanlanscianense* are recorded by provincial standards.

*Polygonatum* is a diverse and widely distributed plant. It is widely distributed across the temperate Northern Hemisphere. *Flora of China* records that it is distributed in many provinces of China, including Heilongjiang, Jilin, Liaoning, Hebei, Shanxi, Shaanxi, Nei Mongol, Ningxia, Gansu, Henan, Shandong, Anhui, Zhejiang, and Sichuan. The rhizome morphology of *Polygonatum* plants is also diverse, with *P. sibiricum* having a “Jitou‐type” and an atypical “Jitou‐type.” *P. cyrtonema* has three types, “Jiang‐type,” “Cylinder‐type,” and “Baiji‐type,” with variations in the composition of the rhizomes of the different types (Hu et al. 2022). In short, the overlapping geographical distribution of *Polygonatum* plants and the diverse morphology of the rhizomes of the medicinal parts make them difficult to identify. Phyllotaxis was usually considered as an important basis for distinguishing plants of the genus (Xia et al. 2022). Previous studies have indicated that species of *Polygonatum* can be divided into alternate leaf types and verticillate leaf types according to the phyllotaxis characteristics; for example, *P. cyrtonema*, *P. filipes* , and *P. odoratum* are alternate leaf types, and *P. sibiricum* , *P. kingianum*, and *P. zanlanscianense* are verticillate leaf types. However, it is extremely difficult to accurately identify the species by morphological features such as phyllotaxis. Establishing an effective identification method to distinguish between the medicinal species of *Polygonatum* is necessary.

DNA barcoding and molecular markers have been used for accurate species delimitation and phylogenetic relationship inference in *Polygonatum* (Jiao et al. 2018; Lee et al. 2021). However, species identification using traditional DNA barcoding techniques is limited and does not allow for the accurate identification of closely related species (Newmaster et al. 2008). The chloroplast genome sequence has been proposed as a superbarcode for species authentication (Li et al. 2015). The chloroplast genomes of medicinal plants such as *Atractylodes* (Wang et al. 2021), *Gentiana* (Zhao et al. 2022), *Peucedanum* (Sun et al. 2023), and *Tripterygium* (Xu et al. 2024) have been studied one after another, and their intragenus evolutionary relationships have been explored. Studies of the chloroplast genomes of *Polygonatum* spp. have also been reported. The genus *Polygonatum* is a monophyletic group comprising three sections (sect. *Sibirica*, sect. *Polygonatum*, and sect. *Verticillata*) (Xia et al. 2022). *Heteropolygonatum*, *Disporopsis*, *Maianthemum*, and *Disporum* are sister groups to *Polygonatum* within Polygonateae (Wang et al. 2022). During the long evolutionary process of the genus, the leaf type of this group evolved from verticillate leaves to alternate leaves. The construction of a phylogenetic tree based on the chloroplast genome showed that *P. kingianum* can be clearly distinguished from other species in the verticillate leaf group, providing a reliable means for the accurate identification of *P. kingianum* (Shi et al. 2023). However, these studies did not focus on sequencing or analyzing the chloroplast genomes of medicinal plants of the genus *Polygonatum*.

Consequently, we sequenced and analyzed the whole chloroplast genomes of six medicinal species of *Polygonatum* to enrich the understanding of genome characteristics, screen mutational hotspots and SSRs for authentication of *Polygonatum* medicinal plants, and elucidate the phylogenetic relationship of *Polygonatum* medicinal plants.

## 2 Materials and Methods

### 2.1 Plant Materials Collection and DNA Extraction

This study collected six medicinal plants of *Polygonatum* from different regions (Figure S1, Table S1). The species was confirmed and identified, and all voucher specimens were stored at the Chinese Materia Medica Resource Center, Anhui University of Chinese Medicine (Hefei, China).

Healthy and fresh leaves were chosen to extract the complete genomic DNA using a plant DNA mini kit (Plant DNA Kit D3485, Omega Bio‐Tek, Guangzhou, China). The purity, integrity, and concentration of the DNA were checked using a NanoDrop 2000 spectrophotometer (Thermo Scientific, Wilmington, DE, USA) and 1.0% (w/v) agarose gel electrophoresis (Wu et al. 2021). The concentration of DNA samples that meet the requirements of chloroplast genome sequencing is ≥ 20 ng/μL; the total amount of samples is ≥ 100 ng; OD_260/280_ = 1.8–2.2, and high‐quality DNA was used to construct gene libraries (Zhu et al. 2018).

### 2.2 Chloroplast DNA Sequencing, Assembly, and Annotation

Genesky Biotechnologies Inc. (Shanghai, China) was commissioned to use Illumina HiSeq 4000 to randomly sequence the chloroplast genomes of each DNA sample from *Polygonatum* plants. Genomic DNA was fragmented after quality control and the adaptor was ligated to construct the library. To obtain high‐quality sequencing data and improve the accuracy of subsequent bioinformatic analyses, quality control, and filtering of the original offline data must be performed. FastQC v0.11.8 software and R v3.6.1 were used to assess the quality of the raw sequencing data, and Kraken2 v2.0.9 software was used to identify chloroplast sequences in the sequencing data. According to the reference near‐source species, metaSPAdes v3.13.0 software (Nurk et al. 2017) was used for genome assembly. The chloroplast genomes were annotated using CPGAVAS2 software (http://47.96.249.172:16019/analyzer/home) (Shi et al. 2019). GenBank files were drawn into a gene circle map using GeSeq (https://chlorobox.mpimp‐golm.mpg.de/geseq.html) (Tillich et al. 2017). The sequence data and gene annotation information were uploaded to the National Center for Biotechnology Information (NCBI) database.

### 2.3 Repeat Sequence and Codon Usage Analysis

SSRs were detected for genomes using the online software MISA v2.1 (Beier et al. 2017) (https://webblast.ipk‐gatersleben.de/misa/), with the minimum repeat parameters set at ten repeat units for mononucleotides, five repeat units for dinucleotides, four repeat units for trinucleotides, three repeat units for tetranucleotides, pentanucleotides, and hexanucleotides (Wang et al. 2022). Forward, palindromic, reverse, and complementary repeats were predicted using the REPuter v1.0 (https://bibiserv.cebitec.uni‐bielefeld.de/reputer), the parameters are set to hamming distance = 3, maximum computed repeats = 5, 000 bp, and minimal repeat size = 30 bp (Kurtz et al. 2001).

Codon usage of the genomes was investigated using the relative synonymous codon usage (RSCU) module in the Python CAI package. The RSCU value represents relative synonymous codon usage. For RSCU = 1, codon usage is without preference; RSCU > 1, codon usage frequency is higher than expected; and RSCU < 1, codon usage frequency is lower than expected (Sharp and Li 1986). Microsoft Office Excel 2016 and TBtools v2.041 (Chen et al. 2020) were used to convert statistical data into visual graphs.

### 2.4 Genome Comparison, Hotspot Identification, and Ka/Ks Calculation

Using CPJSdraw software (Li et al. 2023), we detected the inverted repeat (IR) boundary regions by comparing the locations of the coding genes. Sequence alignment of the whole chloroplast genome was performed using the online tool mVISTA v2.0 (Fernández‐Jiménez et al. 2021) (http://genome.lbl.gov/vista/index.shtml) in shuffle‐LAGAN mode. DnaSP v6 software (Rozas et al. 2017) was used to calculate the nucleotide diversity based on sliding window analysis, setting the window length to 600 bp and the step size to 200 bp. To investigate the presence of selective pressure on the chloroplast protein‐coding genes among *Polygonatum*, we used *P. zanlanscianense* as a reference, and the coding sequences were used to calculate Ka/Ks values using KaKs_Calculator2 (Wang et al. 2010).

### 2.5 Phylogenetic Tree Construction of *Polygonatum* Medicinal Plants

The reported chloroplast genome sequences of *Polygonatum* species were downloaded from NCBI for phylogenetic analysis, setting *Dioscorea aspersa* and *Dioscorea alata* as outgroups (Table S15). A total of 59 chloroplast complete sequences were aligned using MAFFT v7.017 (Katoh and Standley 2013) and trimmed using TrimAL v1.2rev57 (Capella‐Gutiérrez, Silla‐Martínez, and Gabaldón 2009). The best‐fit model according to the Bayesian information criterion was K3Pu+F+I+I+R4, which was calculated using ModelFinder v2.2.0 (Kalyaanamoorthy et al. 2017). An IQ‐TREE (Nguyen et al. 2015) phylogenetic tree was constructed based on the whole chloroplast sequences using the PhyloSuite v1.2.3 platform (Zhang et al. 2020). Also, use MrBayes v3.2.7 to generate a BI tree. The tree is displayed on the iTOL (Letunic and Bork 2021) website (https://itol.embl.de/).

## 3 Results

### 3.1 Basic Characteristics of Chloroplast Genomes

**FIGURE 1:**
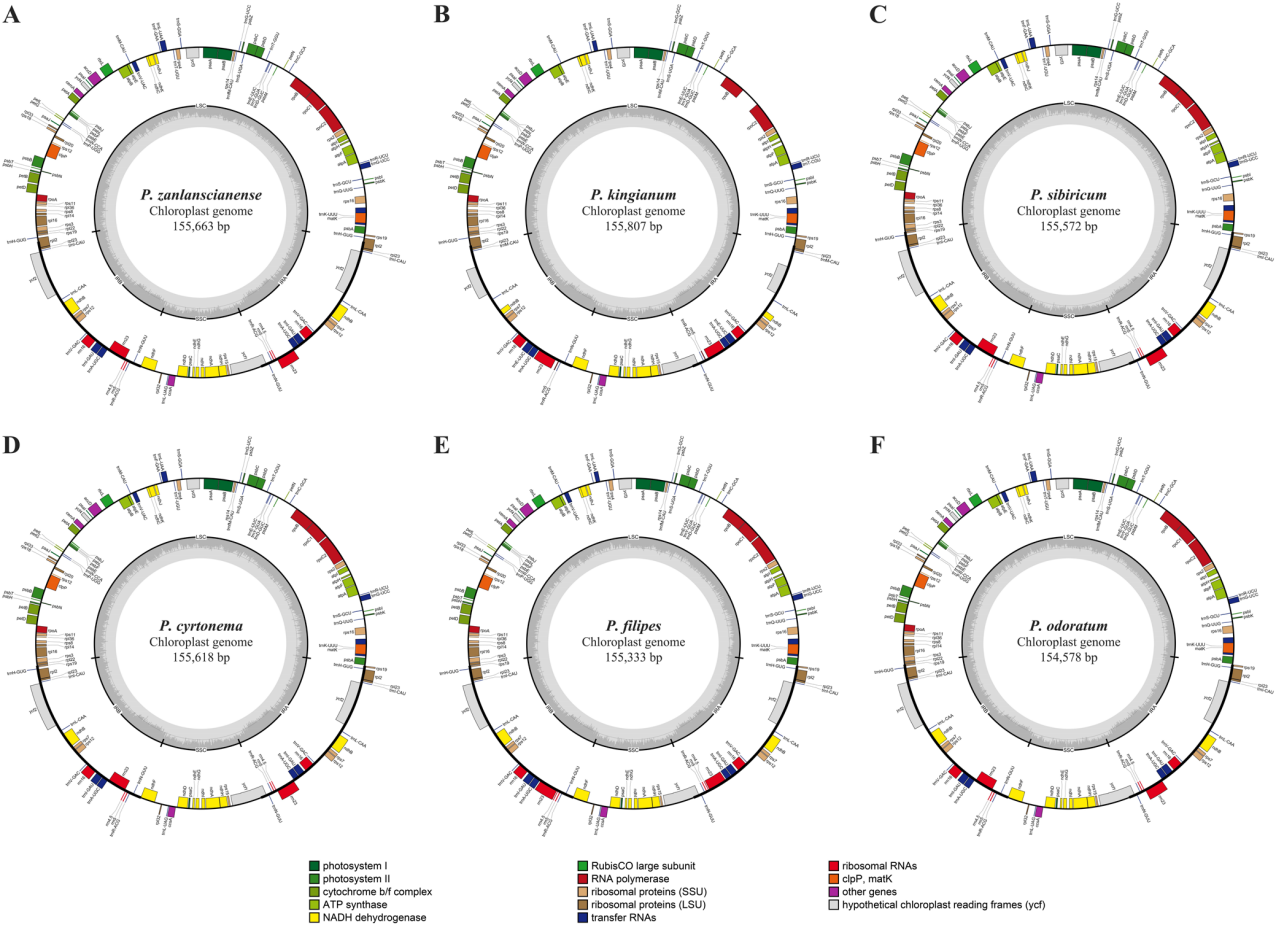
Circular maps for chloroplast genomes of six medicinal plants of *Polygonatum*. (A) *P. zanlanscianense*, (B) *P. kingianum*, (C) *P. sibiricum* , (D) *P. cyrtonema*, (E) *P. filipes* , and (F) *P. odoratum*. Genes inside and outside the loop are transcribed in a clockwise and counterclockwise direction. Genes with diverse functions are represented using different colors.

This study obtained the complete chloroplast genome sequences of six medicinal plants of *Polygonatum*: *P. zanlanscianense*, *P. kingianum*, *P. sibiricum* , *P. cyrtonema*, *P. filipes* , and *P. odoratum*. The length of the complete chloroplast genome sequences ranged from 154,578 bp (*P. odoratum*) to 155,807 bp (*P. kingianum*), with an average length of 155,429 bp. The *Polygonatum* chloroplast genome has a typical quadripartite structure, including large single‐copy (LSC), small single‐copy (SSC), and a pair of IR regions. The length of the LSC ranged from 83,527 bp (*P. odoratum*) to 84,626 bp (*P. kingianum*). In the SSC regions, the chloroplast genome of *P. sibiricum* was the shortest (18, 415 bp), and *P. kingianum* was the longest (18, 529 bp). The IR lengths ranged from 26, 297 bp for *P. odoratum* to 26, 415 bp for *P. zanlanscianense*. GC content was unevenly distributed across the four parts of the chloroplast genome. The total GC content of the chloroplast genome was 37.7%, and the GC contents in the LSC, SSC, and IR regions were 35.7%–35.8%, 31.5%–31.6%, and 43%, respectively (Figure 1, Table 1).

**TABLE 1:** Basic chloroplast characteristics of six medicinal plants of *Polygonatum*.

Characteristics	*Polygonatum zanlanscianense*	*Polygonatum kingianum*	*Polygonatum sibiricum*	*Polygonatum cyrtonema*	*Polygonatum filipes*	*Polygonatum odoratum*
Total size (bp)	155,663	155,807	155,572	155,618	155,333	154,578
LSC length (bp)	84,417	84,626	84,475	84,451	84,279	83,527
IRa length (bp)	26,415	26,326	26,341	26,371	26,300	26,297
IRb length (bp)	26,415	26,326	26,341	26,371	26,300	26,297
SSC length (bp)	18,416	18,529	18,415	18,425	18,454	18,457
Total genes	131	127	131	131	130	131
Protein coding genes	85	84	85	85	84	85
tRNA genes	38	37	38	38	38	38
rRNA genes	8	6	8	8	8	8
Overall GC content (%)	37.7	37.7	37.7	37.7	37.7	37.7
GC content in LSC (%)	35.7	35.7	35.7	35.8	35.7	35.8
GC content in IRa (%)	43	43	43	43	43	43
GC content in IRb (%)	43	43	43	43	43	43
GC content in SSC (%)	31.6	31.5	31.6	31.6	31.6	31.6

Differences were observed in the number of chloroplast genome genes among the six *Polygonatum* species. *P. kingianum* and *P. filipes* encoded 127 and 130 genes, respectively, whereas the other species encoded 131 genes. These genes comprised 84–85 protein‐coding genes, 37–38 transfer RNA genes, and 6–8 ribosomal RNA genes. The IR regions included seven protein‐coding genes (*rpl2*, *rpl23*, *rps12*, *rps19*, *rps7*, *ndhB*, and *ycf2*), 11 tRNA genes (*trnG‐UCC*, *trnE‐UUC*, *trnM‐CAU*, *trnH‐GUG*, *trnI‐CAU*, *trnL‐CAA*, *trnV‐GAC*, *trnI‐GAU*, *trnA‐UGC*, *trnR‐ACG*, and *trnN‐GUU*), and all four rRNA‐coding genes. A total of 22 genes in the chloroplast genomes of the six *Polygonatum* species contained introns *ycf3* and *clpP*, each containing two introns. *rps12*, *trnK‐UUA*, *rps16*, *trnG‐UCC*, *trnT‐CGU*, *atpF*, *rpoC1*, *trnL‐UAA*, *trnV‐UAC*, *petB*, *petD*, *rpl16*, *rpl2*, *ndhB*, *trnI‐GAU*, *trnE‐UUC*, *trnA‐UGC*, and *ndhA* contained a single intron. *trnT‐CGU* was annotated only in *P. kingianum*, which lacked *rrn4.5*, *rpoC1* and *trnV‐UAC*. The *ndhB* gene of *P. kingianum* did not contain introns. The *ycf2* and *ycf1* genes of *P. zanlanscianense* also contained two introns; the *ycf2* gene of *P. sibiricum* and *P. cyrtonema* contained one intron, and *P. kingianum*, *P. filipes*, and *P. odoratum* had no introns. Gene and intron losses occurred during the evolution of the six medicinal plants of *Polygonatum* (Table 2).

**TABLE 2:** List of genes found in the chloroplast genomes of six medicinal plants of *Polygonatum*.

Gene category	Gene name
Ribosomal protein (LSU)	*rpl33*, *rpl20*, *rpl36*, *rpl14*, rpl16*, *rpl22*, rpl2* ^,(×2)^, *rpl23* ^(×2)^, *rpl32*
Ribosomal protein (SSU)	rps12* ^,(×2)^, rps16*, *rps2*, *rps14*, *rps4*, *rps18*, *rps11*, *rps8*, *rps3*, *rps19* ^(×2)^, *rps7* ^(×2)^, *rps15*
RNA polymerase	*rpoA*, *rpoB*, rpoC1*, *rpoC2*
Protease	clpP*
Maturase	*matK*
Ribosomal RNA	*rrn16* ^(×2)^, *rrn23* ^(×2)^, *rrn5* ^(×2)^, *rrn4.5* ^(×2)^
Transfer RNA	trnK‐UUU*, *trnQ‐UUG*, *trnS‐GCU*, trnT‐CGU*, trnG‐UCC*^,(×2)^, *trnR‐UCU*, *trnC‐GCA*, *trnD‐GUC*, *trnY‐GUA*, trnE‐UUC* ^,(×2)^, *trnT‐GGU*, *trnS‐UGA*, *trnG‐GCC*, *trnM‐CAU* ^(×2)^, *trnfM‐CAU*, *trnS‐GGA*, *trnT‐UGU*, trnL‐UAA*, *trnF‐GAA*, trnV‐UAC*, *trnW‐CCA*, *trnP‐UGG*, *trnH‐GUG* ^(×2)^, *trnI‐CAU* ^(×2)^, *trnL‐CAA* ^(×2)^, *trnV‐GAC* ^(×2)^, trnI‐GAU*^,(×2)^, trnA‐UGC*^,(×2)^, *trnR‐ACG* ^(×2)^, *trnN‐GUU* ^(×2)^, *trnL‐UAG*
Photosystem I	*psaB*, *psaA*, *psaI*, *psaJ*, *psaC*
Photosystem II	*psbA*, *psbK*, *psbI*, *psbM*, *psbD*, *psbC*, *psbZ*, *psbJ*, *psbL*, *psbF*, *psbE*, *psbB*, *psbT*, *psbN*, *psbH*
NADH dehydrogenase	*ndhJ*, *ndhK*, *ndhC*, ndhB*^,(×2)^, *ndhF*, *ndhD*, *ndhE*, *ndhG*, *ndhI*, ndhA*, *ndhH*
ATP synthase	*atpA*, atpF*, *atpH*, *atpI*, *atpE*, *atpB*
Cytochrome b/f complex	*petN*, *petA*, *petL*, *petG*, petB*, petD*
Hypothetical chloroplast reading frames (*ycf*)	ycf3*, ycf4, ycf2*^,(×2)^, ycf1*
Rubisco large subunit	*rbcL*
Other gene	*accD*, *cemA*, *ccsA*

*Note:* * Indicates the intron‐containing genes, ^(×2)^ indicates that the gene has two copies.

### 3.2 Simple Sequence Repeats and Interspersed Repeats Sequences Analysis

**FIGURE 2:**
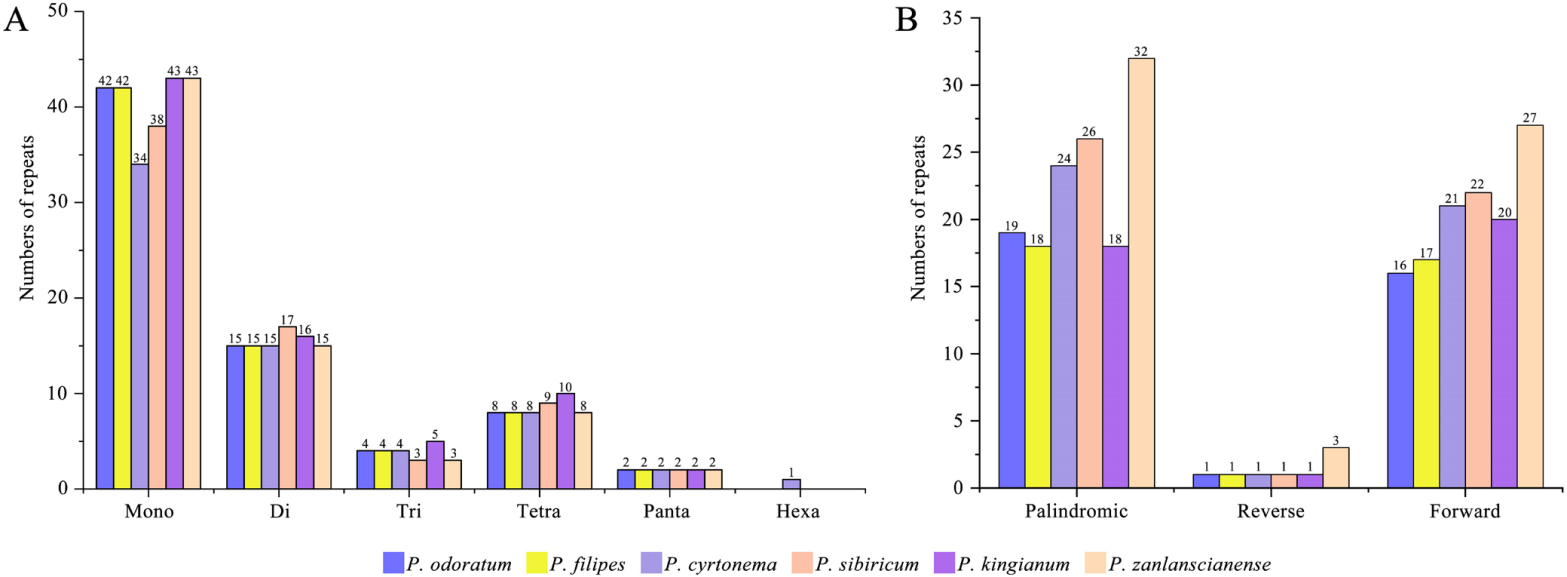
SSRs (A) and long repetitive sequences (B) analysis of chloroplast genomes of six *Polygonatum* species.

The MISA program was used to identify the SSRs in our study. The total number of SSR sites in the six *Polygonatum* chloroplast genomes was 64 (*P. cyrtonema*)–76 (*P. kingianum*), comprising mononucleotide, dinucleotide, trinucleotide, tetranucleotide, and pentanucleotide repeats; only hexanucleotide repeats were found in *P. cyrtonema* (Figure 2A). Among the SSR sites, the number of mononucleotide repeats was the highest, totaling 242. A/T repeat motifs accounted for the largest proportion, ranging from 97.56% to 100%, and *P. kingianum*, *P. filipes* , and *P. odoratum* had C/G mononucleotide repeat motifs. A total of 93 dinucleotide repeats were found, with AT/TA accounting for 80%. Trinucleotide repeat sequences were constant in *P. odoratum*, *P. filipes* , and *P. cyrtonema*, whereas ATT and ATA were absent in the other three *Polygonatum* species. The tetranucleotide and pentanucleotide repeat sequences in the chloroplast genomes of six *Polygonatum* species were similar. Among them, only *P. sibiricum* had AAAT tetranucleotide repeat motifs, and *P. kingianum* had one more AATA than the other species (Table 3, Tables S2–S7).

**TABLE 3:** Type and amounts of SSRs in the chloroplast genomes of six *Polygonatum* species.

SSR type	Repeat unit	*Polygonatum zanlanscianense*	*Polygonatum kingianum*	*Polygonatum sibiricum*	*Polygonatum cyrtonema*	*Polygonatum filipes*	*Polygonatum odoratum*
Mononucleotide	A/T	43	42	38	34	41	41
	C/G	0	1	0	0	1	1
Dinucleotide	AT/AT	6	6	7	5	6	6
	GA/TC	3	3	3	3	3	3
	TA/TA	6	7	7	7	6	6
Trinucleotide	ATA	0	0	1	1	1	1
	ATT	0	1	0	1	1	1
	CAG	1	1	1	1	1	1
	TAA/TTA	2	3	1	1	1	1
Tetranucleotide	AAAT	0	0	1	0	0	0
	AATA	2	3	2	2	2	2
	AATG/CATT	2	2	2	2	2	2
	ATTG	1	2	1	1	1	1
	GAAT	1	1	1	1	1	1
	TTAA	1	1	1	1	1	1
	TTGA	1	1	1	1	1	1
Pentanucleotide	CGAAA/TTTCG	2	2	2	2	2	2
Hexanucleotide	ATAGTA	0	0	0	1	0	0
Total		71	76	69	64	71	71

A total of 268 repetitive sequences were identified in this study. Palindromic repeats accounted for the largest proportion (51.12%), followed by forward repeats (36.07%) and reverse repeats (2.99%). No complementary repeats were found in the chloroplast genomes of the six *Polygonatum* species (Figure 2B). Among these types of *Polygonatum*, *P. zanlanscianense* had significantly longer repetitive sequences than the other species, with 62. *P. sibiricum* (49), *P. cyrtonema* (46), *P. kingianum* (39), *P. filipes* (36), and *P. odoratum* (36). Most species were mainly distributed in the IR region. Among them, the number and distribution of all repeat types in *P. filipes* and *P. odoratum* were almost the same. The distribution area was mainly in the LSC region and could be distinguished from the others (Tables S8–S13), and the repeat motif size of *Polygonatum* was mainly concentrated in the range of 30–39 bp (Figure S2).

### 3.3 Statistics of Codon Usage

**FIGURE 3:**
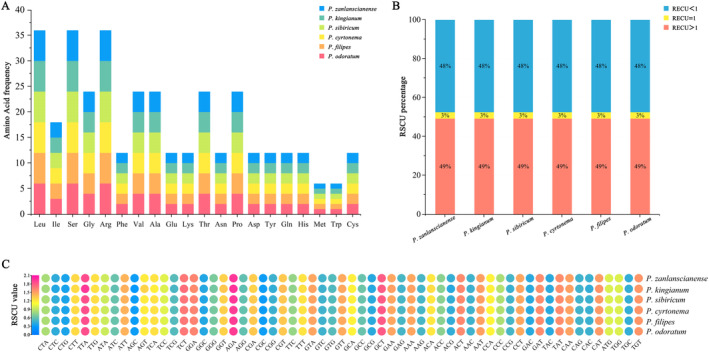
Analysis of amino acids and codon bias among six medicinal plants of *Polygonatum*. (A) Frequency of amino acids in the chloroplast genomes of six *Polygonatum*. (B) RSCU percentage analysis of codons in chloroplast genomes. (C) Heatmap of the RSCU values among six *Polygonatum*.

The total number of codons in the chloroplast genome of the six medicinal plants of *Polygonatum* in protein‐coding sequences was 23,381 (*P. kingianum*) to 26,036 (*P. odoratum*), containing 61 codons encoding 20 amino acids (termination codons were not incorporated in the statistics). Amino acids are encoded by 2–6 synonymous codons, most of which are not Met and Trp. Leu was encoded by the highest number of codons, accounting for 10.3%, whereas Cys was encoded by the lowest number of codons, accounting for 1.2% (except *P. kingianum*, accounting for 1.1%). The RSCU value can be used to detect a synonymous codon usage bias. Except for Met and Trp (RSCU = 1), which do not show codon usage bias, most amino acid codons have usage bias. Thirty types of codons were found with RSCU > 1 in the six medicinal plants of *Polygonatum*, of which 28 were A/T‐ending codons. Only the TTG codon encoding Leu and the TCC codon encoding Ser ended with G/C, indicating that A/T bases were preferred and G/C bases were not preferred. A comprehensive analysis of the histogram and heat map of codon usage showed that codon usage of the six species was consistent. The analysis of RSCU values provided data for studying the evolution and gene expression of *Polygonatum* (Figure 3, Table S14).

### 3.4 IR Borders Comparison

**FIGURE 4:**
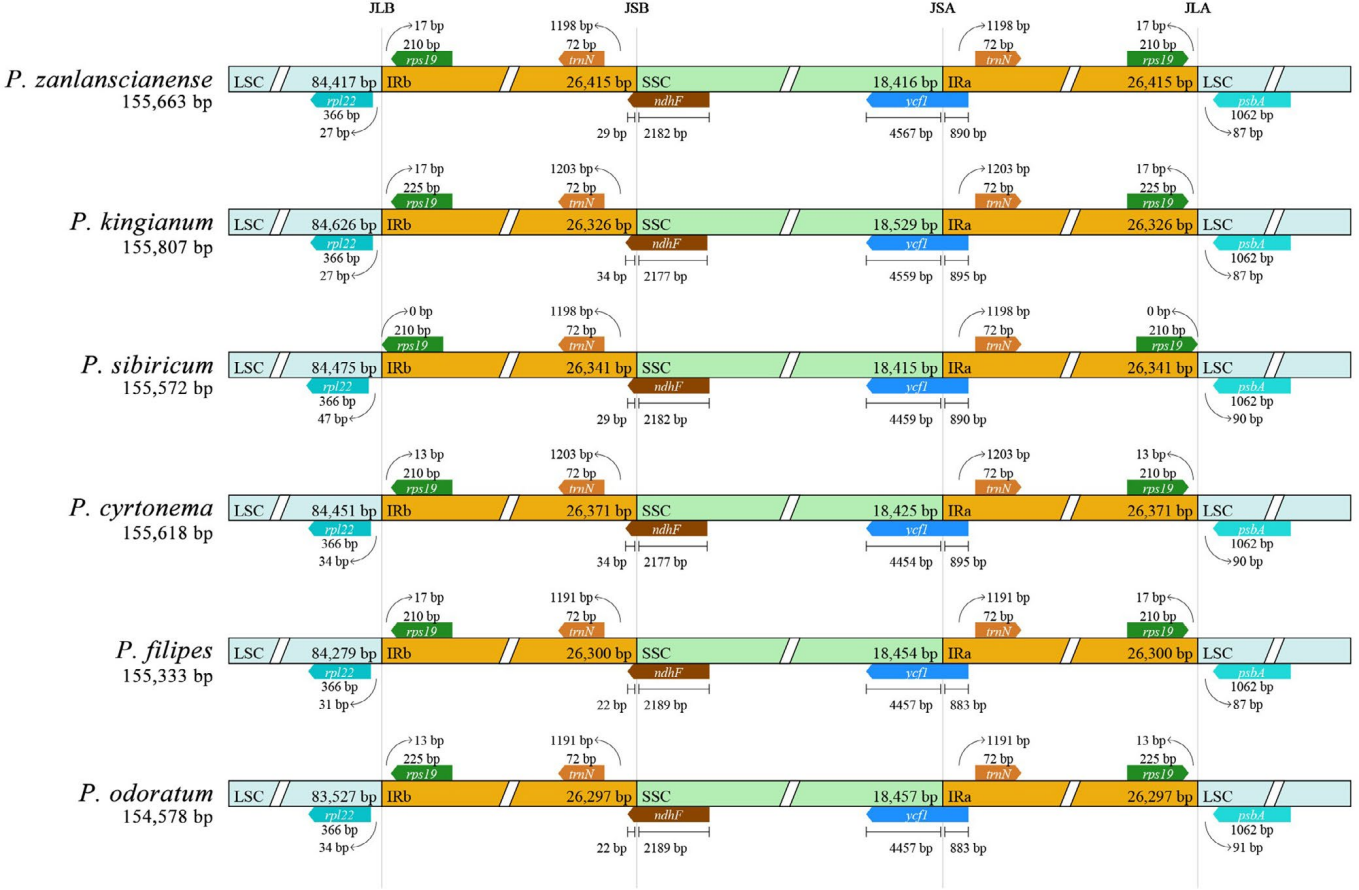
Alignment of LSC, SSC, and IR regions boundary of chloroplast genomes of six medicinal plants of *Polygonatum*.

During plant chloroplast genome evolution, the IR regions are accompanied by contraction and expansion, and some genes enter the IR or SC regions. The IR/SC boundaries and their adjacent genes in the six medicinal plants of *Polygonatum* were compared using CPJSdraw. As shown (Figure 4), the total sequence length and IR region length of the chloroplast genome between species were relatively conserved, and the genotypes of the IR/SC borders were essentially the same. Genes *rpl22*, *rps19*, *trnN*, *ndhF*, *ycf1*, and *psbA* were present at the IR boundaries. The front ends of *rps19* genes of *P. zanlanscianense*, *P. kingianum*, *P. cyrtonema*, *P. filipes* , and *P. odoratum* were 13 or 17 bp away from the IRb boundary, whereas in *P. sibiricum* , the *rps19* gene front ends coincided with the LSC/IRb boundary. In the LSC/IRa boundary, the end of the *rps19* gene of *P. sibiricum* coincides with the LSC/IRa boundary and is also different from other species. *rpl22* was completely situated in the LSC region and was 27–34 bp away from the LSC/IRb boundary; *P. sibiricum* was 47 bp. In six medicinal plants of *Polygonatum*, the *ndhF* gene was prolonged to the IR by 22–34 bp. The *ycf1* gene spans the junction between the SSC and IRa. The *pbsA* gene is located downstream of the junction of LSC and IRa, 87–91 bp from the boundary.

### 3.5 Sequence Divergence and High Variation Regions Analyses

**FIGURE 5:**
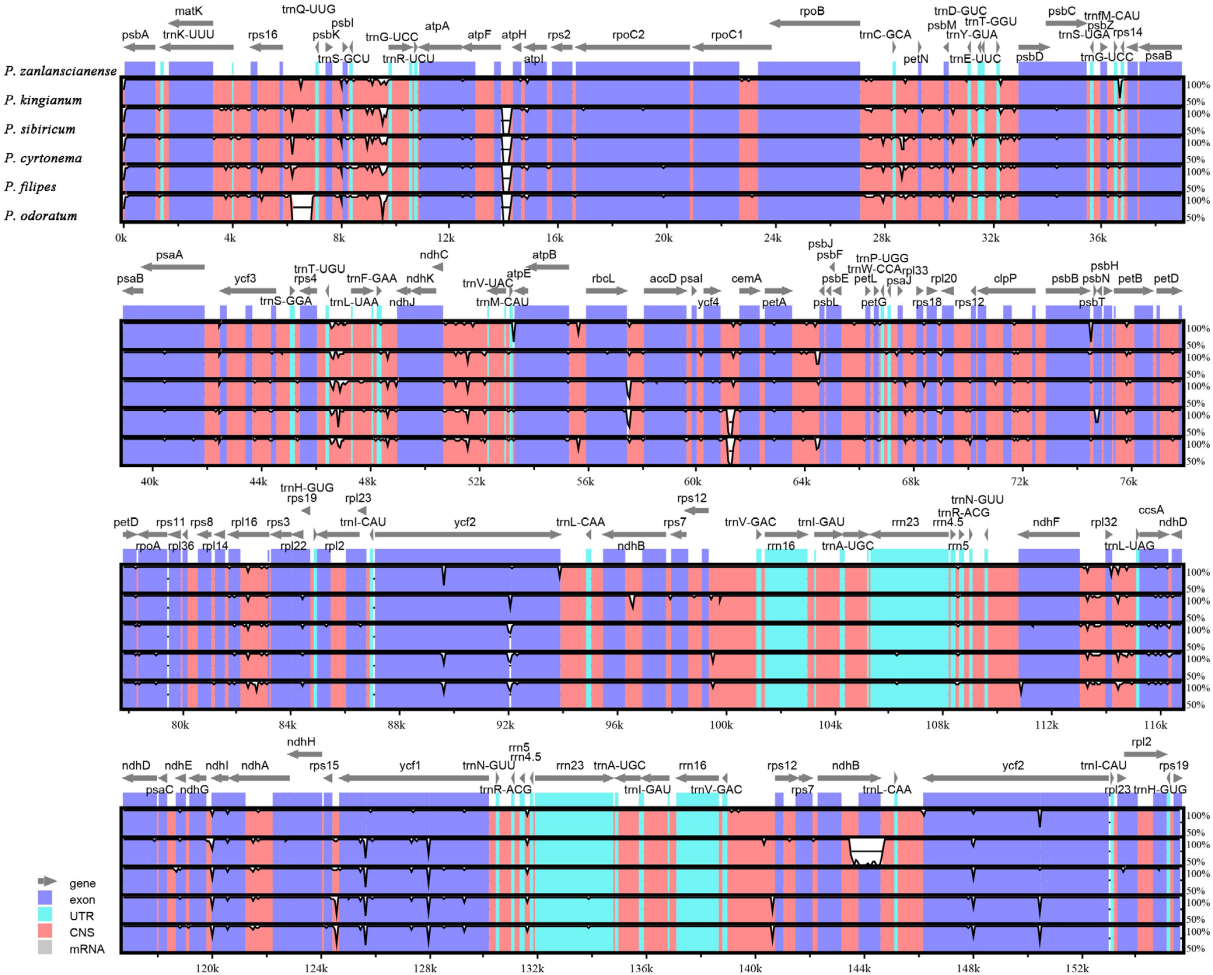
Sequence alignment among six *Polygonatum*, with *P. zanlanscianense* as a reference. The *y*‐axis represents the percent identity within 50%–100%. Genome regions are color coded as genes (gray arrow), protein coding (purple), RNA coding genes (blue), and non‐coding sequences (red).

**FIGURE 6:**
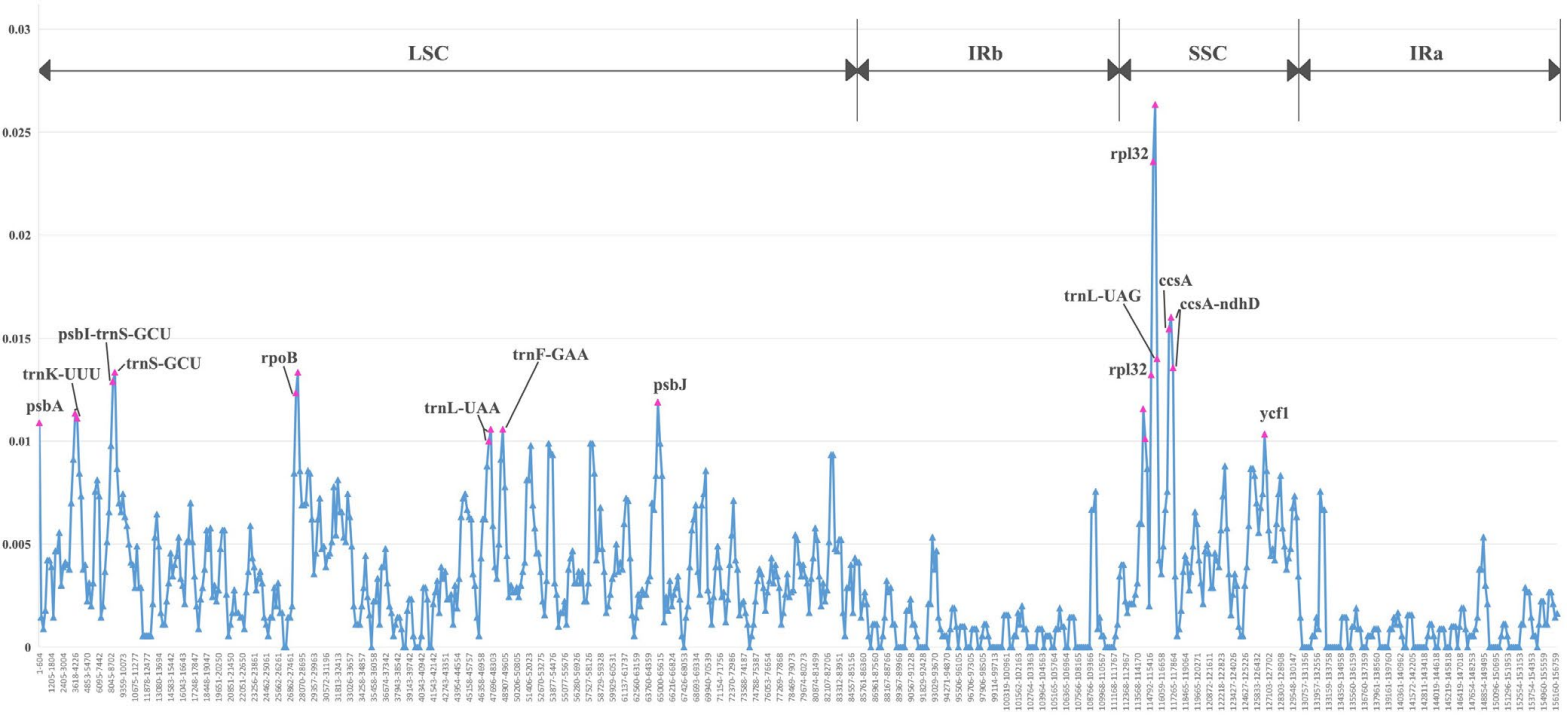
Sliding window analysis of the whole chloroplast genomes of six medicinal plants of *Polygonatum* for nucleotide diversity (Pi). *X*‐axis: Position of the window. *Y*‐axis: Nucleotide diversity of each window. Red signs are the selected high variation regions.

The mVISTA online tool was used to globally align the chloroplast genomes of these *Polygonatum* species, with *P. zanlanscianense* as the reference, and the sequence differences between their genomes were compared (Figure 5). In comparison, the chloroplast genome sequences of six *Polygonatum* species were generally conserved. From the position of sequence differences, the rRNA gene region (blue part) was highly conserved, and the non‐coding region (red part) was more variable than the conserved protein‐coding region (purple part). The variation of the LSC region and the SSR region was greater than that of IR region and the difference was greater in LSC region, followed by SSR region. In addition, DnaSP software was used to determine the nucleotide diversity of the chloroplast genome of six medicinal *Polygonatum* plants and to identify mutation hotspots (Figure 6). The results showed that the Pi values of *Polygonatum* were 0–0.02633 and the high‐variation regions were mainly concentrated in the LSC and SSC regions. Twenty‐one genic regions with high Pi values (Pi ≥ 0.01) were considered hotspots. Among them, 11 genic regions were located in the LSC region, namely *psbA*, *trnK‐UUU*, *psbI‐trnS‐GCU*, *trnS‐GCU*, *rpoB*, *trnL‐UAA*, *trnF‐GAA*, and *psbJ*; among them, 10 genic regions were located in the SSC region, namely *rpl32*, *trnL‐UAG*, *ccsA*, *ccsA‐ndhD*, and *ycf1*. These hotspots provide a reference for the subsequent molecular identification of *Polygonatum* medicinal plants to identify potential chloroplast DNA barcodes.

### 3.6 Selective Pressure Analysis

We calculated the Ka/Ks values of *Polygonatum* protein‐coding genes (PCGs) based on KaKs_Calculator2. The genes subject to positive selection are *matK*, *ndhA*, *petB*, and *ycf2*, with a maximum Ka/Ks value of 1.41. The genes that were positively selected can be categorized into five groups according to their functions: (1) maturation enzyme gene *matK*; (2) photosynthetic system genes *ndhA* and *petB*; and (4) the *ycf2* gene with unknown function. The remaining genes, all with Ka/Ks values less than 1, were subjected to purifying selection, with *atpI* being the lowest at 0.048 (Figure S3).

### 3.7 Phylogenetic Analysis

**FIGURE 7:**
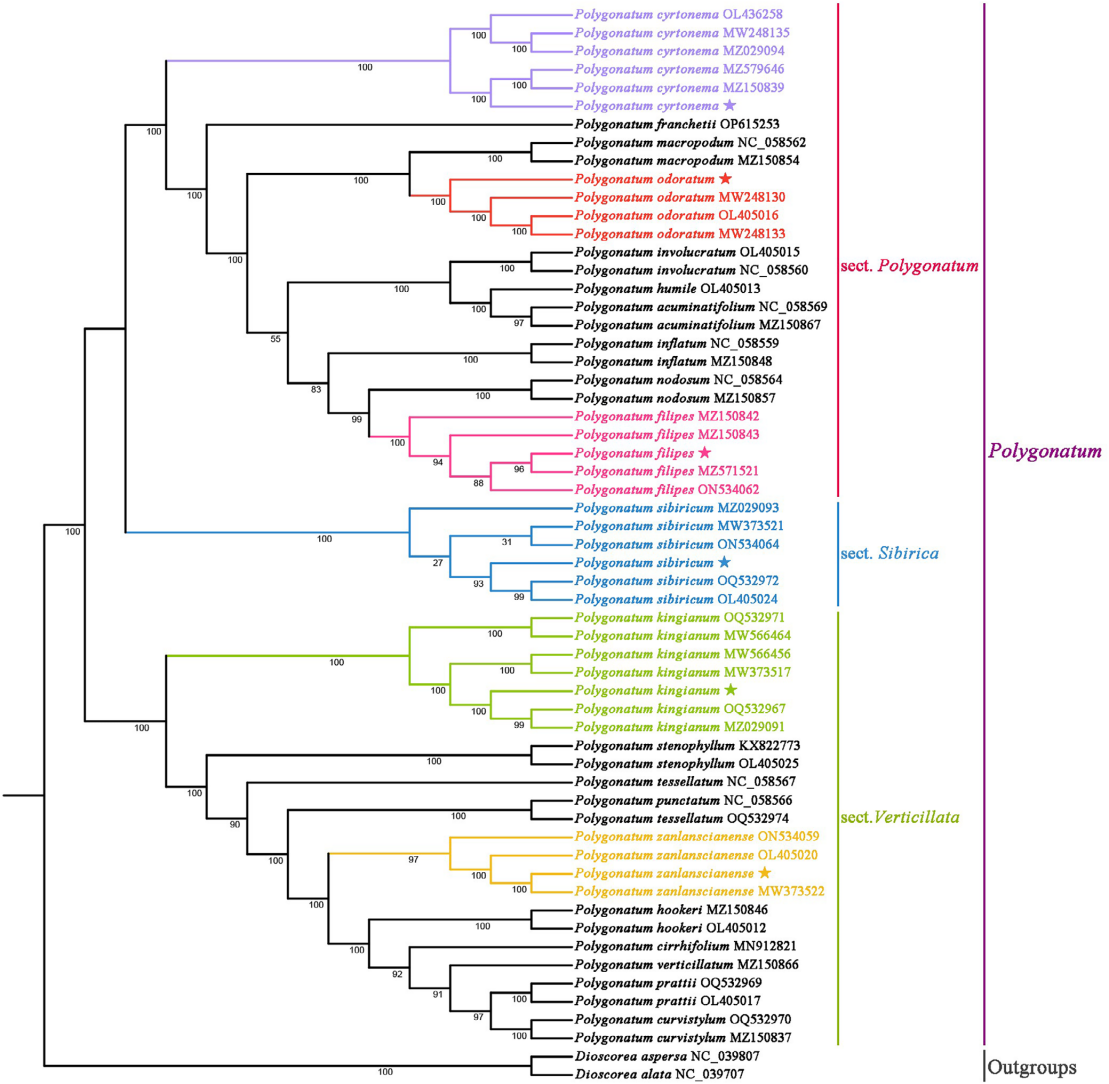
Phylogenetic tree constructed with the whole sequences of the chloroplast genomes of the above 59 species.

Phylogenetic trees were constructed using the maximum likelihood (ML) and Bayesian inference (BI) approaches based on 59 complete chloroplast genome sequences to illustrate the genetic relationships of these species (Figure 7, Figure S4). In the phylogenetic trees, all plants of *Polygonatum* were clustered on one large branch, confirming the monophyly of this genus. *Polygonatum* was divided into three sections: sect. *Polygonatum*, sect. *Sibirica*, and sect. *Verticill*ata, and sect. *Polygonatum* + sect. *Sibirica* were exhibited a sister relationship with sect. *Verticillata* (BS = 100%, BI = 1.0). *P. cyrtonema*, *P. odoratum*, and *P. filipes* clustered on the sect. *Polygonatum* branch (BS, BI = 100%, 1.0, respectively), while *P. kingianum* and *P. zanlanscianense* are located on the sect. *Verticillata* branch (BS, BI = 100%, 1.0, respectively). *P. sibiricum* formed a monophyletic group (sect. *Sibirica*), confirming the independence of this section (BS = 100%, BI = 1.0).

## 4 Discussion

### 4.1 Chloroplast Genome Structure Analysis of *P. kingianum*

In the present study, the chloroplast genome sequences of six medicinal plants of *Polygonatum* were analyzed. According to the assembly annotation results, the chloroplast genomes of *Polygonatum*, similar to most angiosperms, exhibit a classical tetrad structure and are closed‐circular double‐stranded DNA molecules (Daniell et al. 2016). The complete genome length ranged from 154,578 to 155,807 bp, and the lengths of the LSC, SSC, and IR regions were relatively conserved, with no obvious contraction or expansion. However, the chloroplast genome of *P. kingianum* is the largest, and its measured genome size is usually above 155,700 bp (Guo et al. 2022; Zhang et al. 2023). *P. zanlanscianense* chloroplast genome was the next largest. *Polygonatum* chloroplast genome encodes 127–131 genes. *P. kingianum* encodes 127 genes, comprising 84 protein‐coding genes, 37 transfer RNA genes, and 6 ribosomal RNA genes. Compared to other species, *P. kingianum* lacks *rpoC1*, *trnV‐UAC*, and *rrn4.5* genes, which is consistent with the results of previous studies (Wang et al. 2022). The length and composition of the chloroplast genome of *P. kingianum* differ from those of other species, which may be related to the limited geographic growth environment of *P. kingianum*.

SSRs are widely distributed in chloroplast genomes and can be used for species identification and genetic diversity analyses (Jiang et al. 2018). A total of 64–76 SSRs are present in the chloroplast genomes of six *Polygonatum* species (Zhang et al. 2023; Yan et al. 2023). The number of SSRs in *Polygonatum* is comparable to that in *Peucedanum* (Liu et al. 2022), *Adonis* (Nyamgerel et al. 2023), and *Paeoniaceae* (Cai et al. 2023). The A/T and AT/TA repeat sequences account for the largest number of mono‐ and dinucleotide repeat sequences, respectively, influencing the overall GC content of genomes (Gichira et al. 2019).

### 4.2 Relatively Conserved Chloroplast Genomes in *Polygonatum*

In the chloroplast genomes of angiosperms, rRNA genes are generally located in the IR region, which to some extent makes the IR region more conserved than the LSC and SSC regions, making the IR region the most conserved region in the chloroplast genome. However, during the evolution of this species, the IR boundary has undergone contraction and expansion, affecting the length of the chloroplast genome (Li et al. 2013; Zhang et al. 2016). The length of the *Pelargonium hortorum* chloroplast genome is 217,942 bp owing to the extreme expansion of its IR region (Chumley et al. 2006); the IR region length of *Pinus thunbergii* is shortened to only 495 bp, and the chloroplast genome length is 119,707 bp (Wakasugi et al. 1994); whereas the IR of *P. hortorum* is generally absent (Liu and Huang 2020). The IR boundary region of the chloroplast genomes of the six *Polygonatum* medicinal plants is relatively conserved and does not undergo significant expansion or contraction (Zhang et al. 2023; Yan et al. 2023). However, the *rps19* gene of *P. sibiricum* overlapped with the LSC/IR boundary and differed from that of the other five species, which was hypothesized to be related to the evolution of *P. sibiricum* .

The results of mVISTA and nucleotide diversity analyses indicated that the chloroplast genomes of the six *Polygonatum* species were highly similar. The highly variable regions of the chloroplast genomes of *Polygonatum* species were mainly concentrated in the LSC and SSC regions, and 21 highly variable Pi‐fragments were screened. Among them, *psbI‐trnS‐GCU*, *psbJ*, *rpl32*, *trnL‐UAG*, *ccsA*, *ndhD*, and *ycf1* were screened as candidate markers. This provides a reference for subsequent molecular identification of *Polygonatum* species to identify potential chloroplast DNA barcodes.

The selective pressure analysis revealed that the *matK*, *ndhA*, *petB*, and *ycf2* genes were subject to positive selection, of which *ndhA* and *petB* were photosynthetic system genes. *Polygonatum* plants are mainly distributed in the understory, thickets, or shady areas of mountain slopes, and adaptation to sunlight stress may be an important genetic basis for the evolution of adaptations at the chloroplast level in *Polygonatum* (Zhang et al. 2023).

### 4.3 Chloroplast Genome Phylogenetic Analysis Supports Three Clades/Groups of *Polygonatum*

The genus *Polygonatum* is rich in germplasm resources and contains various species that are difficult to identify owing to their similar morphology. The classification of this genus has long been controversial and is a concern for taxonomic treatment. The genus *Polygonatum* was first established by Miller (Zhao et al. 2014). Baker (1875) divided *Polygonatum* into three groups, *Alternifolia*, *Verticillata*, and *Oppositifolia*, according to the characteristics of phyllotaxis. However, species of *Polygonatum* differ in morphological characteristics, such as perianth and bracts, in addition to differences in leaf type. *Flora of China* synthesized these characteristics and divided the genus into eight groups. Tamura subdivided the genus into two sections: *Polygonatum* and *Verticillata* (Tamura 1993). To date, botanists have not reached a unified view of the classification of *Polygonatum* species based on their morphological characteristics.

With the development of molecular biology, phylogenetic studies of species based on chloroplast genome sequences have provided novel perspectives for solving plant affinities. Previous phylogenetic studies of *Polygonatum* chloroplast genomes have supported the classification of this genus into three groups: sect. *Verticillata*, sect. *Polygonatum*, and sect. *Sibirica*, where sect. *Verticillata* is a sister species of sect. *Polygonatum* + sect. *Sibirica* (Floden and Schilling 2018). Sect. *Verticillata* contains *Polygonatum* species with verticillate leaf types, *sect*. *Polygonatum* contains alternate leaf types, and *sect*. *Sibirica* usually has only one species, *P. sibiricum* (Qin et al. 2024). Consistent with previous findings, the chloroplast genome phylogenetic tree of *Polygonatum* used in the present study was divided into three clades: sect. *Verticillata*, sect. *Polygonatum*, and sect. *Sibirica*. *P. kingianum* and *P. zanlanscianense* clustered in the verticillate leaf taxon sect. *Verticillata*. *P. cyrtonema*, *P. filipes* , and *P. odoratum* clustered into the alternate‐leaf taxon sect. *Polygonatum*. *P. sibiricum* forms a monophyletic group with reference to the following morphological characters. The leaves, typically arranged in whorls of three to six. The flowers are axillary, pendant, and white; they are borne on arcuate‐deflexed peduncles, usually with two to four flowers. The berries are invariably black, containing four to seven seeds (Meng et al. 2014).

## 5 Conclusions

This study utilized the Illumina Hiseq platform to obtain the complete chloroplast genomes of six medicinal species of the genus *Polygonatum*. The chloroplast genomes were relatively conserved in terms of length, gene content, and genomic structure. A certain pattern was observed in genome length among species. *Polygonatum* spp. with verticillate leaves were larger than those with alternating leaves. The genes encoded by *P. kingianum* appeared to be lost compared to those of the other species. Twenty‐one highly variable loci were screened as markers for the candidate identification of *Polygonatum* species. The phylogeny of the species in the genus was studied based on chloroplast genome sequences, and the results supported the classification of *Polygonatum* into three groups: sect. *Verticillata*, sect. *Polygonatum*, and sect. *Sibirica*. This study provides a basis for the molecular identification, phylogenetic evolution, and genetic diversity of *Polygonatum*.

## Author Contributions

**Jinchen Yao:** conceptualization (equal), data curation (equal), methodology (equal), writing – original draft (equal). **Zhaohuan Zheng:** conceptualization (equal), data curation (equal), software (equal), writing – original draft (equal). **Tao Xu:** formal analysis (equal), investigation (equal), resources (equal), visualization (equal). **Duomei Wang:** methodology (equal), software (equal), supervision (equal), visualization (equal). **Jingzhe Pu:** formal analysis (equal), investigation (equal), validation (equal). **Yazhong Zhang:** conceptualization (equal), funding acquisition (equal), project administration (equal), writing – review and editing (equal). **Liangping Zha:** conceptualization (equal), funding acquisition (equal), project administration (equal), writing – review and editing (equal).

## Consent

The authors have nothing to report.

## Conflicts of Interest

The authors declare no conflicts of interest.

## Supporting information


Appendix S1.

## Data Availability

The datasets analyzed for this study are available in the GenBank repositories; the accession numbers are OQ928151, OQ928152, OQ928153, OQ928154, OQ928155, and OQ928156.
