# Supplementary material for: Chloroplast Genome Sequencing and Comparative Analysis of Six Medicinal Plants of Polygonatum
Source: Ecol Evol. 2025 Jan 10;15(1):e70831. doi: 10.1002/ece3.70831 (PMC11718222; doi:10.1002/ece3.70831)
Supplement: Supplementary file 1 — Appendix S1. [file ECE3-15-e70831-s001.pdf]

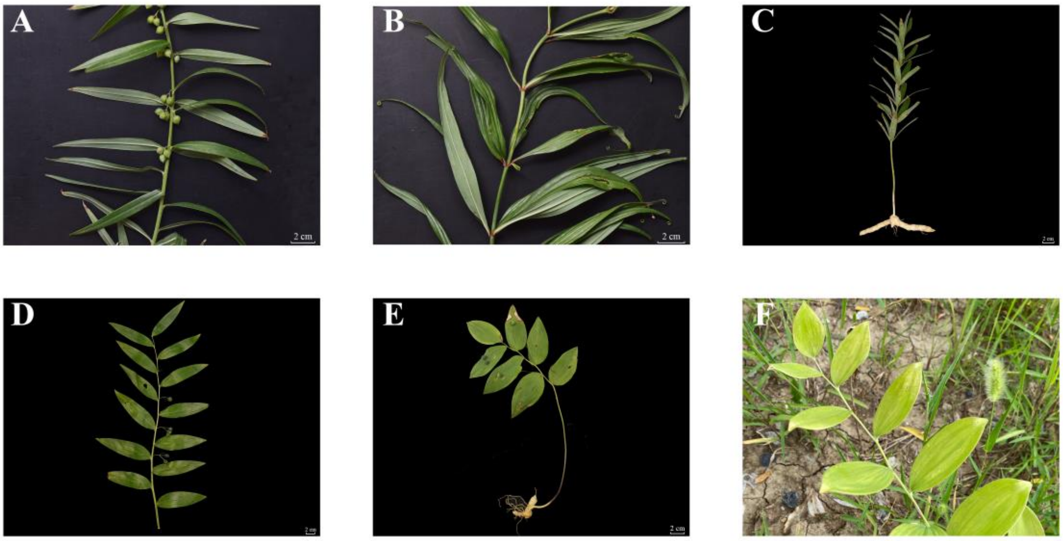

Figure S1 Leaf collection of six *Polygonatum* medicinal plants. (A) *P. zanlanscianense*, (B) *P. kingianum*, (C) *P. sibiricum*, (D) *P. cyrtonema*, and (E) *P. filipes*, (F) *P. odoratum*.

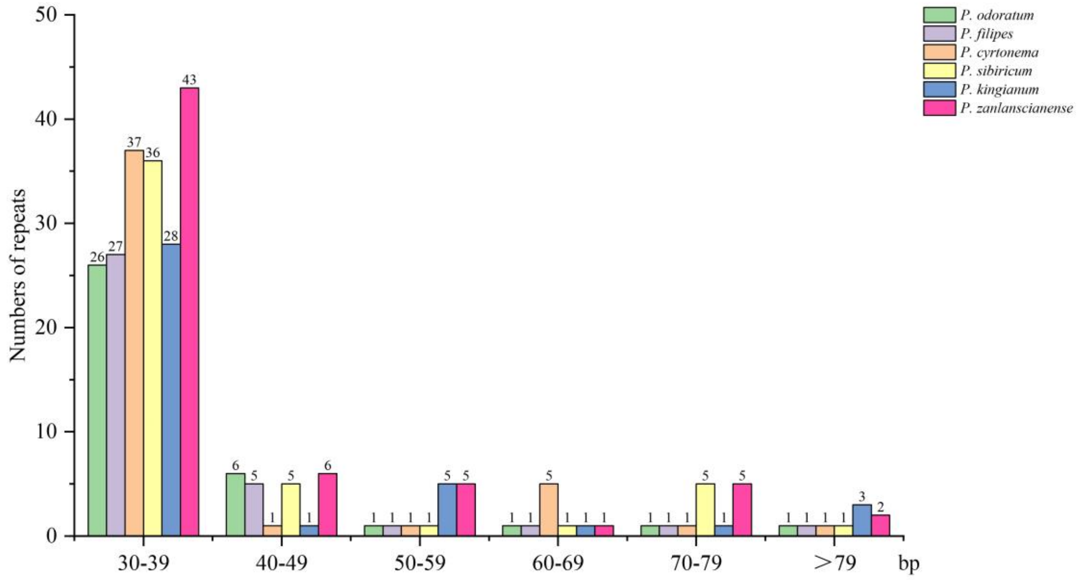

Figure S2 Length of long repetitive sequences in the chloroplast genomes of six *Polygonatum* medicinal plants.

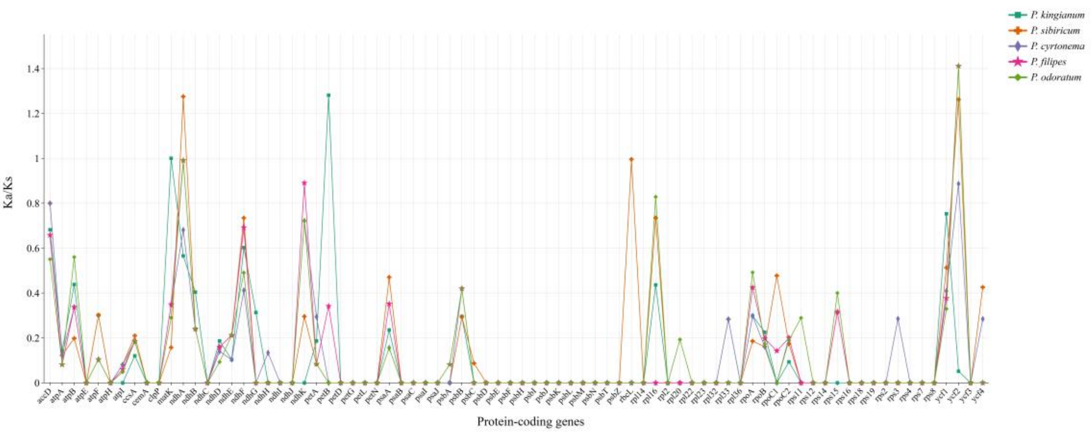

Figure S3 Ka/Ks ratios of PCGs in the chloroplast genomes of *Polygonatum*.

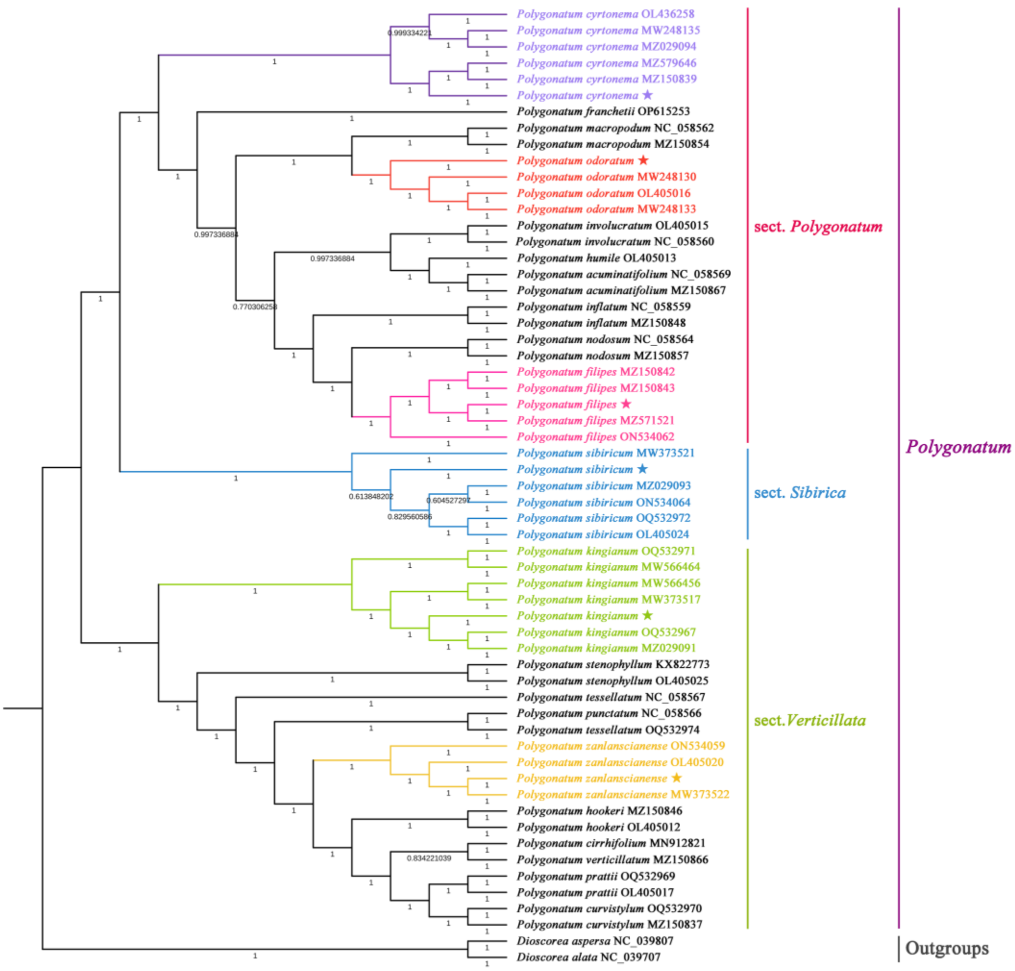

Figure S4 Phylogenetic tree constructed with the whole sequences of the chloroplast genomes of the 59 species using BI methods.

Table S1 Information of the sample collection and Genbank numbers.

Species	Kinds of leaf	Locality	GenBank accession number
P. odoratum	alternate	Hefei, Anhui, China	OQ928151
P. filipes	alternate	Huangshan, Anhui, China	OQ928152
P. cyrtonema	alternate	luan, Anhui, China	OQ928153
P. sibiricum	verticillate	luan, Anhui, China	OQ928156
P. kingianum	verticillate	Puer, Yunnan, China	OQ928154
P. zanlanscianense	verticillate	Huanggang, Hubei, China	OQ928155

Table S2 SSRs in *P. odoratum.*

SSR number	Length (bp)	SSR type	Base and length	Repeat start	Repeat end	Region
1	12	p3	(CAG)4	685	696	LSC
2	11	p1	(A)11	2952	2962	LSC
3	10	p1	(A)10	3573	3582	LSC
4	10	p1	(T)10	4676	4685	LSC
5	10	p1	(T)10	7297	7306	LSC
6	54	c	(T)13attccatacatttaacatttacctatgatg(A)11	7551	7604	LSC
7	108	c	(TA)6aattctatttcattatattatgaattaggatcaagtatgatcaagtcaagttttatttaaataagctgctttctattcttcgccta(T)10	8784	8891	LSC
8	10	p1	(T)10	13154	13163	LSC
9	10	p2	(TA)5	13903	13912	LSC
10	12	p3	(TTA)4	14965	14976	LSC
11	99	c	(T)10aacaaataaacaaataaataattgttcctacggaaccttctgccggaattggccgttgatacacagcccaaaccattca(T)10	15721	15819	LSC
12	117	c	(T)10ccgttcctggtggtatcaaaatgccactgtgtctggatatcttatctgtctctccgggaaaatgaatatctccagaaaagattttcagttcaatac(T)11	17794	17910	LSC
13	10	p2	(AT)5	19279	19288	LSC
14	10	p1	(A)10	21955	21964	LSC
15	95	c	(A)10tgggttcgaaattcgatctcctctctatgaatgagataaagacagaataatcaaaagtagtatagagtttcc(T)13	26613	26707	LSC
16	56	c	(ATA)4aaaacaatactatagctggtgtggtagaaagaac(TA)5	29200	29255	LSC
17	10	p1	(T)10	30460	30469	LSC
18	10	p1	(T)10	31433	31442	LSC
19	10	p1	(T)10	34589	34598	LSC
20	10	p1	(A)10	35440	35449	LSC
21	13	p1	(T)13	41695	41707	LSC
22	16	p1	(A)16	43538	43553	LSC
23	10	p2	(AT)5	45792	45801	LSC
24	92	c	(A)11gaaagaagaaagaatatcgacccttctagtatttcaaatca	46230	46321	LSC

			cgctgtaaaaacaaaagggaag ggagac(AT)6			
25	11	p1	(T)11	47092	47102	LSC
26	10	p1	(T)10	54451	54460	LSC
27	10	p2	(TA)5	54818	54827	LSC
28	12	p4	(ATTG)3	56709	56720	LSC
29	22	c	(AT)5ca(AT)5	57126	57147	LSC
30	10	p1	(A)10	58792	58801	LSC
31	12	p2	(TA)6	60416	60427	LSC
32	10	p2	(TC)5	60721	60730	LSC
33	12	p4	(AATG)3	61340	61351	LSC
34	82	c	(T)10gattgacctcctccttgc aggaggtcaaattcaagttgcaa ttcaactgtgttttgttaag(T)11	65737	65818	LSC
35	104	c	(TA)5ataatattctattaatatta attagaattacaacaaaatattta gaaatggaatttctaattagtaac ttctattgatattga(T)11	67011	67114	LSC
36	12	p2	(AT)6	67465	67476	LSC
37	13	p1	(T)13	68016	68028	LSC
38	11	p1	(A)11	69849	69859	LSC
39	11	p1	(T)11	70105	70115	LSC
40	11	p1	(T)11	70385	70395	LSC
41	12	p1	(A)12	70917	70928	LSC
42	15	p1	(T)15	73578	73592	LSC
43	11	p1	(G)11	75210	75220	LSC
44	12	p4	(AATA)3	81519	81530	LSC
45	10	p2	(GA)5	89278	89287	IRb
46	12	p4	(GAAT)3	104012	104023	IRb
47	15	p5	(CGAAA)3	107905	107919	IRb
48	10	p1	(A)10	112450	112459	SSC
49	10	p1	(T)10	112991	113000	SSC
50	121	c	(TTAA)4tacttaatgatacta agaaaagtatacaaatt gttagaaaaattacttaaatttagt aattgaattgtgata catatgaaatcctattta(T)15	113633	113753	SSC
51	11	p1	(A)11	114867	114877	SSC
52	12	p4	(AATA)3	115681	115692	SSC
53	12	p4	(TTGA)3	117768	117779	SSC
54	15	p1	(T)15	118844	118858	SSC
55	10	p1	(A)10	120826	120835	SSC
56	15	p3	(ATT)5	123589	123603	SSC
57	10	p1	(A)10	127112	127121	SSC

58	15	p5	(TTTCG)3	130187	130201	IRa
59	12	p4	(CATT)3	134082	134093	IRa
60	10	p2	(TC)5	148819	148828	IRa

Table S3 SSRs in *P. filipes.*

SSR number	Length (bp)	SSR type	Base and length	Repeat start	Repeat end	Region
1	12	p3	(CAG)4	681	692	LSC
2	11	p1	(A)11	2948	2958	LSC
3	24	c	(A)10t(A)13	3558	3581	LSC
4	11	p1	(T)11	4693	4703	LSC
5	11	p1	(T)11	7996	8006	LSC
6	54	c	(T)11atattccatacatttaacatttacctatgatg(A)11	8251	8304	LSC
7	111	c	(TA)7aattctatttcattatattatgaattaggatcaagtatgatcaagtcaagttttatttaaataagctgctttctattcttcgccta(T)11	9511	9621	LSC
8	10	p1	(T)10	10574	10583	LSC
9	10	p1	(T)10	13886	13895	LSC
10	10	p2	(TA)5	14635	14644	LSC
11	12	p3	(TTA)4	15697	15708	LSC
12	10	p1	(T)10	16453	16462	LSC
13	117	c	(T)10ccgttcctggtggtatcaaaatgccactgtgtctggatatcttatctgtctctccgggaaaatgaatatctccagaaaagattttcagttcaatac(T)11	18525	18641	LSC
14	10	p2	(AT)5	20010	20019	LSC
15	10	p1	(A)10	22686	22695	LSC
16	11	p1	(T)11	23077	23087	LSC
17	96	c	(A)11tgggttcgaaattcgatctcctctctatgaatgagataaagacagaataatcaaaagtagtatagagtttcc(T)13	27345	27440	LSC
18	10	p1	(A)10	28688	28697	LSC
19	11	p1	(T)11	29670	29680	LSC
20	56	c	(ATA)4aaaacaatactatagctggtgtggtagaaagaac(TA)5	29894	29949	LSC
21	10	p1	(T)10	32126	32135	LSC
22	10	p1	(T)10	35287	35296	LSC
23	10	p1	(A)10	36138	36147	LSC
24	14	p1	(T)14	42393	42406	LSC
25	13	p1	(A)13	44237	44249	LSC
26	10	p2	(AT)5	46488	46497	LSC
27	94	c	(A)13gaaagaagaaagaatatcgacccttctagtatttcaaatcgcgctgtaaaaacaaaagggaagggagac(AT)6	46955	47048	LSC
28	12	p1	(T)12	47819	47830	LSC
29	10	p2	(TA)5	55545	55554	LSC
30	12	p4	(ATTG)3	57436	57447	LSC
31	22	c	(AT)5ca(AT)5	57853	57874	LSC
32	10	p1	(A)10	59519	59528	LSC

33	12	p2	(TA)6	61144	61155	LSC
34	10	p2	(TC)5	61449	61458	LSC
35	12	p4	(AATG)3	62068	62079	LSC
36	10	p1	(T)10	66480	66489	LSC
37	10	p2	(TA)5	67754	67763	LSC
38	14	p2	(AT)7	68227	68240	LSC
39	11	p1	(T)11	68780	68790	LSC
40	10	p1	(A)10	70602	70611	LSC
41	11	p1	(T)11	70857	70867	LSC
42	10	p1	(T)10	71137	71146	LSC
43	53	c	(A)10cataagttttggctcccttccatatccct(A)14	71630	71682	LSC
44	11	p1	(T)11	74338	74348	LSC
45	10	p1	(G)10	75973	75982	LSC
46	12	p4	(AATA)3	82255	82266	LSC
47	10	p2	(GA)5	90034	90043	IRb
48	12	p4	(GAAT)3	104768	104779	IRb
49	15	p5	(CGAAA)3	108660	108674	IRb
50	10	p1	(A)10	113204	113213	SSC
51	117	c	(TTAA)4tacttaatgatactaagaaaagtatacaaa ttgttagaaaaattatttaaatttagtaattgaattgtg atacatatgaaatcctattta(T)11	114390	114506	SSC
52	11	p1	(A)11	115619	115629	SSC
53	12	p4	(AATA)3	116433	116444	SSC
54	12	p4	(TTGA)3	118520	118531	SSC
55	14	p1	(T)14	119596	119609	SSC
56	11	p1	(A)11	121577	121587	SSC
57	15	p3	(ATT)5	124341	124355	SSC
58	10	p1	(A)10	127864	127873	SSC
59	15	p5	(TTTCG)3	130939	130953	IRa
60	12	p4	(CATT)3	134833	134844	IRa
61	10	p2	(TC)5	149570	149579	IRa

Table S4 SSRs in *P. cyrtonema.*

SSR number	Length (bp)	SSR type	Base and length	Repeat start	Repeat end	Region
1	12	p3	(CAG)4	684	695	LSC
2	11	p1	(A)11	2951	2961	LSC
3	12	p1	(A)12	4543	4554	LSC
4	53	c	(T)11atattccatacatttaacatttacctatgatg(A)10	8221	8273	LSC
5	12	p1	(A)12	8456	8467	LSC
6	46	c	(TA)6tttaatttgattatatattg(TA)7	9437	9482	LSC
7	10	p2	(TA)5	14585	14594	LSC
8	12	p3	(TTA)4	15647	15658	LSC
9	10	p1	(T)10	16404	16413	LSC
10	117	c	(T)10ccgttcctggtggtatcaaaatgccactgtgtctggatatcttatctgtctctccgggaaaatgaatatctccagaaaagattttcagttcaatac(T)11	18476	18592	LSC
11	10	p2	(AT)5	19961	19970	LSC
12	10	p1	(A)10	22637	22646	LSC
13	13	p1	(T)13	23028	23040	LSC
14	94	c	(A)10tgggttcgaaattcgatctcctctctatgaatgagataaagacagaagaatcaaaagtagtatagagtttcc(T)12	27298	27391	LSC
15	12	p1	(A)12	27801	27812	LSC
16	56	c	(ATA)4aaaacaatactatagctggtgtggtagaaagaac(TA)5	29892	29947	LSC
17	12	p1	(T)12	31163	31174	LSC
18	118	c	(T)10gtttgacccgttaccagatatattctggaatatgagtcataggacaattcagggttccaatgtttattttatatagtatgtttattttat(ATAGTA)3	32564	32681	LSC
19	10	p1	(T)10	35318	35327	LSC
20	12	p1	(A)12	36169	36180	LSC
21	11	p1	(T)11	42426	42436	LSC
22	15	p1	(A)15	44267	44281	LSC
23	12	p2	(AT)6	47045	47056	LSC
24	10	p1	(T)10	47819	47828	LSC
25	10	p2	(TA)5	55545	55554	LSC
26	12	p4	(ATTG)3	57436	57447	LSC
27	22	c	(AT)5ca(AT)5	57854	57875	LSC
28	10	p1	(A)10	59520	59529	LSC
29	12	p2	(TA)6	61345	61356	LSC
30	86	c	(A)10tgaaaaaaaagaaagcattgatttccctcccatatcttgcatctatagtatttttgccctggtggg(TC)5	61575	61660	LSC
31	12	p4	(AATG)3	62270	62281	LSC
32	10	p1	(T)10	66746	66755	LSC
33	10	p2	(TA)5	67954	67963	LSC
34	16	p2	(AT)8	68406	68421	LSC

35	10	p1	(T)10	68969	68978	LSC
36	16	p1	(T)16	71039	71054	LSC
37	10	p1	(A)10	71855	71864	LSC
38	10	p1	(A)10	75334	75343	LSC
39	12	p4	(AATA)3	82425	82436	LSC
40	10	p2	(GA)5	90238	90247	IRb
41	12	p4	(GAAT)3	105000	105011	IRb
42	15	p5	(CGAAA)3	108891	108905	IRb
43	10	p1	(T)10	113018	113027	SSC
44	15	p1	(A)15	113574	113588	SSC
45	118	c	(TTAA)4tacttaatgatactaagaaaagtatacaaattgttagaaaaattacttaaatttagtaattgaattgtgatacatatgaaatcctattta(T)12	114621	114738	SSC
46	11	p1	(A)11	115852	115862	SSC
47	12	p4	(AATA)3	116660	116671	SSC
48	12	p4	(TTGA)3	118740	118751	SSC
49	15	p1	(T)15	119816	119830	SSC
50	10	p1	(A)10	121801	121810	SSC
51	12	p3	(ATT)4	124561	124572	SSC
52	10	p1	(A)10	128081	128090	SSC
53	15	p5	(TTTCG)3	131165	131179	IRa
54	12	p4	(CATT)3	135058	135069	IRa
55	10	p2	(TC)5	149823	149832	IRa

Table S5 SSRs in *P. sibiricum.*

SSR number	Length (bp)	SSR type	Base and length	Repeat start	Repeat end	Region
1	12	p3	(CAG)4	684	695	LSC
2	11	p1	(A)11	2951	2961	LSC
3	17	p1	(A)17	3561	3577	LSC
4	10	p1	(T)10	6720	6729	LSC
5	54	c	(T)13attccatacatttaacatttacctatgatg(A)11	8216	8269	LSC
6	48	c	(TA)7tttaatttgattatatattg(TA)7	9416	9463	LSC
7	10	p1	(T)10	9927	9936	LSC
8	11	p1	(T)11	10505	10515	LSC
9	10	p1	(T)10	13825	13834	LSC
10	10	p2	(TA)5	14574	14583	LSC
11	12	p3	(TTA)4	15635	15646	LSC
12	11	p1	(T)11	16391	16401	LSC
13	117	c	(T)10ccgttcctggtggtatcaaaatgccactgtgtctggatatcttatctgtctctccgggaaaatgaatatctccagaaaagattttcagttcaatac(T)11	18464	18580	LSC
14	10	p2	(AT)5	19952	19961	LSC
15	10	p1	(A)10	22628	22637	LSC
16	10	p1	(T)10	23019	23028	LSC
17	10	p1	(T)10	27370	27379	LSC
18	10	p1	(A)10	27782	27791	LSC
19	56	c	(ATA)4aaaacaatactatagctggtgtggtagaaagaac(TA)5	29889	29944	LSC
20	10	p1	(T)10	31149	31158	LSC
21	11	p1	(T)11	32550	32560	LSC
22	14	p1	(T)14	35282	35295	LSC
23	11	p1	(A)11	36132	36142	LSC
24	11	p1	(T)11	42389	42399	LSC
25	14	p1	(A)14	44230	44243	LSC
26	27	c	(AT)5aactaga(AT)5	46483	46509	LSC
27	91	c	(A)14gaagaaagaatatcgacccttctagtatttcaaatcgcgctgtaaaaacaaaagggaagggagac(AT)6	46982	47072	LSC
28	10	p2	(TA)5	55575	55584	LSC
29	43	c	(ATTG)3caattaaactcggcccaatcc(T)10	57466	57508	LSC
30	22	c	(AT)5ca(AT)5	57884	57905	LSC
31	10	p2	(TA)5	61380	61389	LSC
32	10	p2	(TC)5	61684	61693	LSC
33	12	p4	(AATG)3	62303	62314	LSC
34	10	p2	(TA)5	67952	67961	LSC
35	16	p2	(AT)8	68403	68418	LSC
36	14	p1	(T)14	68958	68971	LSC

37	11	p1	(A)11	70778	70788	LSC
38	10	p1	(T)10	71036	71045	LSC
39	13	p1	(T)13	71315	71327	LSC
40	54	c	(A)13cataagttttggctcccttccatatccct(A)12	71812	71865	LSC
41	11	p1	(T)11	74515	74525	LSC
42	10	p1	(T)10	75988	75997	LSC
43	12	p4	(AAAT)3	81667	81678	LSC
44	12	p4	(AATA)3	82435	82446	LSC
45	11	p1	(T)11	82920	82930	LSC
46	10	p2	(GA)5	90252	90261	IRb
47	12	p4	(GAAT)3	104998	105009	IRb
48	15	p5	(CGAAA)3	108890	108904	IRb
49	11	p1	(A)11	113435	113445	SSC
50	10	p1	(A)10	113573	113582	SSC
51	117	c	(TTAA)4tacttaatgatactaagaaaagtatccaaat tgttaga aaaattacttaaatttagtaattgaattgtgatacatatgaa atcctat tta(T)11	114599	114715	SSC
52	11	p1	(A)11	115829	115839	SSC
53	12	p4	(AATA)3	116643	116654	SSC
54	12	p4	(TTGA)3	118730	118741	SSC
55	10	p1	(A)10	121780	121789	SSC
56	10	p1	(A)10	128060	128069	SSC
57	15	p5	(TTTCG)3	131144	131158	IRa
58	12	p4	(CATT)3	135038	135049	IRa
59	10	p2	(TC)5	149787	149796	IRa

Table S6 SSRs in *P. kingianum.*

SSR number	Length (bp)	SSR type	Base and length	Repeat start	Repeat end	Region
1	12	p3	(CAG)4	681	692	LSC
2	11	p1	(A)11	2948	2958	LSC
3	10	p1	(A)10	3564	3573	LSC
4	10	p1	(A)10	4534	4543	LSC
5	10	p1	(T)10	7975	7984	LSC
6	57	c	(T)13gatattccatgcatttaacatttacctatgatg(A)11	8229	8285	LSC
7	52	c	(A)10cttcgttttctttttttaattatttattag(TAA)4	8478	8529	LSC
8	15	p3	(TAA)5	8976	8990	LSC
9	54	c	(TA)9tttaatttgattatatattg(TA)8	9514	9567	LSC
10	12	p1	(T)12	13935	13946	LSC
11	10	p1	(A)10	14599	14608	LSC
12	10	p2	(TA)5	14933	14942	LSC
13	12	p3	(TTA)4	15994	16005	LSC
14	10	p1	(T)10	16750	16759	LSC
15	117	c	(T)10ccgttcctggtggtatcaaaatgccactgtgtctggatatcttatctgtctctccgggaaaatgaatatctccagaaaagattttcagttcaatac(T)11	18821	18937	LSC
16	10	p2	(AT)5	20306	20315	LSC
17	15	p1	(A)15	22982	22996	LSC
18	11	p1	(T)11	23378	23388	LSC
19	12	p1	(T)12	27731	27742	LSC
20	11	p1	(A)11	28145	28155	LSC
21	12	p1	(A)12	29045	29056	LSC
22	10	p2	(TA)5	30292	30301	LSC
23	10	p1	(T)10	31531	31540	LSC
24	10	p1	(T)10	32504	32513	LSC
25	11	p1	(T)11	32933	32943	LSC
26	10	p1	(A)10	36507	36516	LSC
27	11	p1	(T)11	42696	42706	LSC
28	15	p1	(A)15	44537	44551	LSC
29	10	p2	(AT)5	46790	46799	LSC
30	92	c	(A)15gaagaaagaatatcgaccctctagtatttcaaatcacgctgtaaaaacaaaagggaagggagac(AT)6	47274	47365	LSC
31	10	p1	(T)10	48136	48145	LSC
32	10	p1	(A)10	52464	52473	LSC
33	10	p2	(TA)5	55807	55816	LSC

34	12	p4	(ATTG)3	57699	57710	LSC
35	22	c	(AT)5ca(AT)5	58116	58137	LSC
36	11	p1	(A)11	59782	59792	LSC
37	10	p1	(T)10	61132	61141	LSC
38	10	p2	(TA)5	61597	61606	LSC
39	10	p2	(TC)5	61900	61909	LSC
40	12	p4	(AATG)3	62519	62530	LSC
41	12	p4	(ATTG)3	66062	66073	LSC
42	12	p1	(T)12	67004	67015	LSC
43	10	p2	(TA)5	68190	68199	LSC
44	12	p2	(AT)6	68641	68652	LSC
45	12	p1	(T)12	69192	69203	LSC
46	10	p1	(T)10	71264	71273	LSC
47	10	p1	(T)10	71543	71552	LSC
48	10	p1	(A)10	72038	72047	LSC
49	10	p1	(A)10	75503	75512	LSC
50	12	p4	(AATA)3	82603	82614	LSC
51	10	p1	(T)10	83092	83101	LSC
52	10	p2	(GA)5	90378	90387	IRb
53	10	p1	(T)10	100797	100806	IRb
54	12	p4	(GAAT)3	105130	105141	IRb
55	15	p5	(CGAAA)3	109021	109035	IRb
56	10	p1	(C)10	113869	113878	SSC
57	116	c	(TTAA)4tacttaatgatactaagaaaagtatccaaattgttagaaaaattacttcaatttagtaattgaattgtgatacatatgaaatcc(TATT)4	114733	114848	SSC
58	11	p1	(A)11	115969	115979	SSC
59	12	p4	(AATA)3	116783	116794	SSC
60	12	p4	(TTGA)3	118870	118881	SSC
61	10	p1	(A)10	121933	121942	SSC
62	12	p3	(ATT)4	124693	124704	SSC
63	11	p1	(T)11	125890	125900	SSC
64	10	p1	(T)10	126105	126114	SSC
65	10	p1	(A)10	128315	128324	SSC
66	15	p5	(TTTCG)3	131399	131413	IRa
67	12	p4	(CATT)3	135292	135303	IRa
68	10	p1	(A)10	139628	139637	IRa
69	10	p2	(TC)5	150047	150056	IRa

Table S7 SSRs in *P. zanlanscianense.*

SSR number	Length (bp)	SSR type	Base and length	Repeat start	Repeat end	Region
1	12	p3	(CAG)4	681	692	LSC
2	11	p1	(A)11	2948	2958	LSC
3	13	p1	(A)13	3564	3576	LSC
4	11	p1	(A)11	4538	4548	LSC
5	12	p1	(A)12	8269	8280	LSC
6	10	p1	(T)10	8430	8439	LSC
7	15	p3	(TAA)5	8965	8979	LSC
8	58	c	(TA)6at(TA)5tttcatttgattatatattg(TA)7	9496	9553	LSC
9	10	p1	(T)10	12426	12435	LSC
10	10	p1	(T)10	13928	13937	LSC
11	12	p3	(TTA)4	15765	15776	LSC
12	10	p1	(T)10	16521	16530	LSC
13	117	c	(T)10ccgttcctggtggtatcaaaatgccactgtgtctggatatcttatctgtctctccgggaaaatgaatatctccagaaaagattttcagttcaatac(T)11	18591	18707	LSC
14	10	p2	(AT)5	20076	20085	LSC
15	11	p1	(A)11	22752	22762	LSC
16	15	p1	(T)15	27495	27509	LSC
17	10	p1	(A)10	27912	27921	LSC
18	10	p1	(A)10	28805	28814	LSC
19	10	p2	(TA)5	30050	30059	LSC
20	11	p1	(T)11	31289	31299	LSC
21	10	p1	(T)10	32263	32272	LSC
22	10	p1	(T)10	32683	32692	LSC
23	10	p1	(T)10	35420	35429	LSC
24	11	p1	(A)11	36256	36266	LSC
25	12	p1	(T)12	42505	42516	LSC
26	15	p1	(A)15	44346	44360	LSC
27	10	p2	(AT)5	46596	46605	LSC
28	91	c	(A)14gaagaaagaatatcgacccttctagtatttcaaatcacgctgtaaaacaaaagggaagggagac(AT)6	47102	47192	LSC
29	11	p1	(T)11	47963	47973	LSC
30	12	p1	(T)12	55266	55277	LSC
31	10	p2	(TA)5	55635	55644	LSC
32	12	p4	(ATTG)3	57480	57491	LSC
33	22	c	(AT)5ca(AT)5	57896	57917	LSC
34	12	p1	(A)12	59562	59573	LSC

35	10	p1	(T)10	60913	60922	LSC
36	86	c	(A)10tgaaaaaaaagaaagcatt<br>gatttccctcccatatcttgcatctata<br>gtatttttgccctggtggg(TC)5	61596	61681	LSC
37	12	p4	(AATG)3	62291	62302	LSC
38	10	p1	(A)10	67800	67809	LSC
39	10	p2	(TA)5	67915	67924	LSC
40	14	p2	(AT)7	68366	68379	LSC
41	10	p1	(T)10	70987	70996	LSC
42	10	p1	(T)10	71267	71276	LSC
43	10	p1	(A)10	71761	71770	LSC
44	10	p1	(T)10	74458	74467	LSC
45	10	p1	(A)10	75278	75287	LSC
46	16	p1	(A)16	75797	75812	LSC
47	12	p4	(AATA)3	82386	82397	LSC
48	11	p1	(T)11	82880	82890	LSC
49	11	p1	(T)11	83087	83097	LSC
50	10	p2	(GA)5	90211	90220	IRb
51	12	p4	(GAAT)3	105015	105026	IRb
52	15	p5	(CGAAA)3	108906	108920	IRb
53	11	p1	(A)11	113281	113291	SSC
54	119	c	(TTAA)4tacttaatgatactaag<br>aaaagtatccaaattgttagaaaaatt<br>acttaaatttagtaattgaattgtgata<br>catatgaaatcctattta(T)13	114604	114722	SSC
55	11	p1	(A)11	115836	115846	SSC
56	12	p4	(AATA)3	116650	116661	SSC
57	12	p4	(TTGA)3	118737	118748	SSC
58	10	p1	(T)10	119819	119828	SSC
59	13	p1	(A)13	121689	121701	SSC
60	11	p1	(T)11	125658	125668	SSC
61	10	p1	(A)10	128077	128086	SSC
62	15	p5	(TTTCG)3	131161	131175	IRa
63	12	p4	(CATT)3	135054	135065	IRa
64	10	p2	(TC)5	149861	149870	IRa

Table S8 Long repetitive sequences in *P. odoratum.*

Number	Length (bp)	Repeat type	Repeat start 1	Repeat start 2	Hamming	Region	E-value
1	30	F	8418	8458	-3	LSC;LSC	6.39E-04
2	30	F	42875	98416	-3	LSC;IRb	6.39E-04
3	30	P	42875	139659	-3	LSC;IRa	6.39E-04
4	30	F	86143	86166	-3	IRb;IRb	6.39E-04
5	30	P	86143	151909	-3	IRb;IRa	6.39E-04
6	30	P	86166	151932	-3	IRb;IRa	6.39E-04
7	30	F	151909	151932	-3	IRa;IRa	6.39E-04
8	31	P	63502	63545	0	LSC;LSC	1.46E-09
9	31	P	7648	44334	-1	LSC;LSC	1.36E-07
10	31	F	7648	34731	-2	LSC;LSC	6.10E-06
11	31	F	9768	35701	-3	LSC;LSC	1.77E-04
12	31	P	31256	31271	-3	LSC;LSC	1.77E-04
13	32	P	46769	46769	0	LSC;LSC	3.64E-10
14	32	P	34730	44334	-3	LSC;LSC	4.88E-05
15	33	F	91055	91079	-2	IRb;IRb	4.33E-07
16	33	P	91055	146993	-2	IRb;IRa	4.33E-07
17	33	P	91079	147017	-2	IRb;IRa	4.33E-07
18	33	F	146993	147017	-2	IRa;IRa	4.33E-07
19	33	P	75173	95346	-3	LSC;IRb	1.34E-05
20	33	F	75173	142726	-3	LSC;IRa	1.34E-05
21	33	P	123687	123687	-3	SSC;SSC	1.34E-05
22	34	R	125165	125165	-2	SSC;SSC	1.15E-07
23	39	P	66656	66656	-1	LSC;LSC	2.60E-12
24	39	F	42863	98404	-2	LSC;IRb	1.48E-10
25	39	P	42863	139662	-2	LSC;IRa	1.48E-10
26	39	P	124929	124929	-3	SSC;SSC	5.49E-09
27	40	F	8426	8446	0	LSC;LSC	5.56E-15
28	41	F	88631	88655	-1	IRb;IRb	1.71E-13
29	41	P	88631	149409	-1	IRb;IRa	1.71E-13
30	41	P	88655	149433	-1	IRb;IRa	1.71E-13
31	41	F	149409	149433	-1	IRa;IRa	1.71E-13
32	41	F	37947	40171	-2	LSC;LSC	1.03E-11
33	52	P	29224	29224	0	LSC;LSC	3.31E-22
34	67	F	37915	40139	-3	LSC;LSC	3.99E-25
35	71	F	37907	40131	-3	LSC;LSC	1.86E-27
36	26297	P	83527	128281	0	IRb;IRa	0.00E+00

Table S9 Long repetitive sequences in *P. filipes.*

Number	Length (bp)	Repeat type	Repeat start 1	Repeat start 2	Hamming	Region	E-value
1	30	F	9118	9138	-3	LSC;LSC	6.45E-04
2	30	F	43574	99172	-3	LSC;IRb	6.45E-04
3	30	P	43574	140410	-3	LSC;IRa	6.45E-04
4	30	F	86899	86922	-3	IRb;IRb	6.45E-04
5	30	P	86899	152660	-3	IRb;IRa	6.45E-04
6	30	P	86922	152683	-3	IRb;IRa	6.45E-04
7	30	F	152660	152683	-3	IRa;IRa	6.45E-04
8	31	P	64240	64283	0	LSC;LSC	1.47E-09
9	31	P	8348	45030	-1	LSC;LSC	1.37E-07
10	31	F	8348	35429	-2	LSC;LSC	6.16E-06
11	31	F	3558	44236	-3	LSC;LSC	1.79E-04
12	31	F	10498	36399	-3	LSC;LSC	1.79E-04
13	31	P	31949	31964	-3	LSC;LSC	1.79E-04
14	32	P	47496	47496	0	LSC;LSC	3.68E-10
15	32	F	67776	67799	-2	LSC;LSC	1.64E-06
16	32	P	35428	45030	-3	LSC;LSC	4.93E-05
17	33	F	91811	91835	-2	IRb;IRb	4.37E-07
18	33	P	91811	147744	-2	IRb;IRa	4.37E-07
19	33	P	91835	147768	-2	IRb;IRa	4.37E-07
20	33	F	147744	147768	-2	IRa;IRa	4.37E-07
21	33	P	75936	96102	-3	LSC;IRb	1.35E-05
22	33	F	75936	143477	-3	LSC;IRa	1.35E-05
23	33	P	124439	124439	-3	SSC;SSC	1.35E-05
24	34	R	125917	125917	-2	SSC;SSC	1.16E-07
25	39	F	43562	99160	-2	LSC;IRb	1.50E-10
26	39	P	43562	140413	-2	LSC;IRa	1.50E-10
27	39	P	125681	125681	-3	SSC;SSC	5.54E-09
28	41	F	89387	89411	-1	IRb;IRb	1.73E-13
29	41	P	89387	150160	-1	IRb;IRa	1.73E-13
30	41	P	89411	150184	-1	IRb;IRa	1.73E-13
31	41	F	150160	150184	-1	IRa;IRa	1.73E-13
32	41	F	38645	40869	-2	LSC;LSC	1.04E-11
33	52	P	29918	29918	0	LSC;LSC	3.35E-22
34	67	F	38613	40837	-3	LSC;LSC	4.03E-25
35	71	F	38605	40829	-3	LSC;LSC	1.88E-27
36	26300	P	84279	129033	0	IRb;IRa	0.00E+00

Table S10 Long repetitive sequences in *P. cyrtonema*.

Number	Length (bp)	Repeat type	Repeat start 1	Repeat start 2	Hamming	Region	E-value
1	30	F	89569	89635	-2	IRb;IRb	2.31E-05
2	30	P	89569	150404	-2	IRb;IRa	2.31E-05
3	30	F	89585	89630	-2	IRb;IRb	2.31E-05
4	30	P	89585	150409	-2	IRb;IRa	2.31E-05
5	30	P	89630	150454	-2	IRb;IRa	2.31E-05
6	30	P	89635	150470	-2	IRb;IRa	2.31E-05
7	30	F	150406	150472	-2	IRa;IRa	2.31E-05
8	30	F	9072	9092	-3	LSC;LSC	6.48E-04
9	30	F	43604	99376	-3	LSC;IRb	6.48E-04
10	30	P	43604	140663	-3	LSC;IRa	6.48E-04
11	30	F	87061	87084	-3	IRb;IRb	6.48E-04
12	30	P	87061	152955	-3	IRb;IRa	6.48E-04
13	30	P	87084	152978	-3	IRb;IRa	6.48E-04
14	30	F	89614	89635	-3	IRb;IRb	6.48E-04
15	30	P	89614	150404	-3	IRb;IRa	6.48E-04
16	30	P	89635	150425	-3	IRb;IRa	6.48E-04
17	30	F	150406	150427	-3	IRa;IRa	6.48E-04
18	30	F	152955	152978	-3	IRa;IRa	6.48E-04
19	31	P	64441	64484	0	LSC;LSC	1.48E-09
20	31	P	8317	45062	-1	LSC;LSC	1.37E-07
21	31	F	8317	35461	-2	LSC;LSC	6.18E-06
22	31	F	10453	36432	-3	LSC;LSC	1.79E-04
23	31	P	31961	31976	-3	LSC;LSC	1.79E-04
24	32	P	35460	45062	-3	LSC;LSC	4.94E-05
25	33	F	92015	92039	-2	IRb;IRb	4.39E-07
26	33	P	92015	147997	-2	IRb;IRa	4.39E-07
27	33	P	92039	148021	-2	IRb;IRa	4.39E-07
28	33	F	147997	148021	-2	IRa;IRa	4.39E-07
29	33	P	76103	96306	-3	LSC;IRb	1.36E-05
30	33	F	76103	143730	-3	LSC;IRa	1.36E-05
31	33	P	124656	124656	-3	SSC;SSC	1.36E-05
32	33	P	125901	125901	-3	SSC;SSC	1.36E-05
33	34	P	9408	9408	-2	LSC;LSC	1.17E-07
34	34	R	126134	126134	-2	SSC;SSC	1.17E-07
35	35	F	150440	150485	-1	IRa;IRa	6.06E-10
36	39	F	43592	99364	-2	LSC;IRb	1.50E-10
37	39	P	43592	140666	-2	LSC;IRa	1.50E-10
38	41	F	38678	40902	-2	LSC;LSC	1.04E-11
39	52	P	29916	29916	0	LSC;LSC	3.36E-22
40	62	F	89549	89594	-2	IRb;IRb	5.45E-24
41	62	P	89549	150413	-2	IRb;IRa	5.45E-24

42	62	P	89594	150458	-2	IRb;IRa	5.45E-24
43	62	F	150413	150458	-2	IRa;IRa	5.45E-24
44	67	F	38646	40870	-3	LSC;LSC	4.05E-25
45	71	F	38638	40862	-3	LSC;LSC	1.89E-27
46	26371	P	84451	129247	0	IRb;IRa	0.00E+00

Table S11 Long repetitive sequences in *P. sibiricum.*

Number	Length (bp)	Repeat type	Repeat start 1	Repeat start 2	Hamming	Region	E-value
1	30	F	89586	89649	-2	IRb;IRb	2.31E-05
2	30	P	89586	150368	-2	IRb;IRa	2.31E-05
3	30	P	89649	150431	-2	IRb;IRa	2.31E-05
4	30	F	150370	150433	-2	IRa;IRa	2.31E-05
5	30	F	9079	9099	-3	LSC;LSC	6.47E-04
6	30	F	43567	99377	-3	LSC;IRb	6.47E-04
7	30	P	43567	140640	-3	LSC;IRa	6.47E-04
8	30	F	87078	87101	-3	IRb;IRb	6.47E-04
9	30	P	87078	152916	-3	IRb;IRa	6.47E-04
10	30	P	87101	152939	-3	IRb;IRa	6.47E-04
11	30	F	152916	152939	-3	IRa;IRa	6.47E-04
12	31	P	64465	64508	0	LSC;LSC	1.48E-09
13	31	P	8313	45024	-1	LSC;LSC	1.37E-07
14	31	F	8313	35424	-2	LSC;LSC	6.18E-06
15	31	F	10429	36394	-3	LSC;LSC	1.79E-04
16	31	P	31945	31960	-3	LSC;LSC	1.79E-04
17	32	P	47520	47520	0	LSC;LSC	3.69E-10
18	32	P	35423	45024	-3	LSC;LSC	4.94E-05
19	33	F	89602	89644	-1	IRb;IRb	9.13E-09
20	33	P	89602	150370	-1	IRb;IRa	9.13E-09
21	33	P	89644	150412	-1	IRb;IRa	9.13E-09
22	33	F	89623	89644	-2	IRb;IRb	4.38E-07
23	33	P	89623	150370	-2	IRb;IRa	4.38E-07
24	33	P	89644	150391	-2	IRb;IRa	4.38E-07
25	33	F	92029	92053	-2	IRb;IRb	4.38E-07
26	33	P	92029	147961	-2	IRb;IRa	4.38E-07
27	33	P	92053	147985	-2	IRb;IRa	4.38E-07
28	33	F	147961	147985	-2	IRa;IRa	4.38E-07
29	33	P	76107	96320	-3	LSC;IRb	1.36E-05
30	33	F	76107	143694	-3	LSC;IRa	1.36E-05
31	33	P	124635	124635	-3	SSC;SSC	1.36E-05
32	33	P	125880	125880	-3	SSC;SSC	1.36E-05
33	34	R	126113	126113	-2	SSC;SSC	1.16E-07
34	36	F	150404	150425	0	IRa;IRa	1.44E-12
35	39	F	43555	99365	-2	LSC;IRb	1.50E-10
36	39	P	43555	140643	-2	LSC;IRa	1.50E-10
37	41	F	38641	40865	-2	LSC;LSC	1.04E-11
38	49	F	89586	89628	-2	IRb;IRb	2.27E-16
39	49	P	89586	150370	-2	IRb;IRa	2.27E-16
40	49	P	89628	150412	-2	IRb;IRa	2.27E-16

41	49	F	150370	150412	-2	IRa;IRa	2.27E-16
42	52	P	29913	29913	0	LSC;LSC	3.36E-22
43	67	F	38609	40833	-3	LSC;LSC	4.04E-25
44	70	F	89586	89607	-3	IRb;IRb	7.22E-27
45	70	P	89586	150370	-3	IRb;IRa	7.22E-27
46	70	P	89607	150391	-3	IRb;IRa	7.22E-27
47	70	F	150370	150391	-3	IRa;IRa	7.22E-27
48	71	F	38601	40825	-3	LSC;LSC	1.88E-27
49	26341	P	84475	129231	0	IRb;IRa	0.00E+00

Table S12 Long repetitive sequences in *P. kingianum*.

Number	Length (bp)	Repeat type	Repeat start 1	Repeat start 2	Hamming	Region	E-value
1	30	F	9462	114701	-3	LSC;SSC	6.49E-04
2	30	F	43874	99505	-3	LSC;IRb	6.49E-04
3	30	P	43874	140898	-3	LSC;IRa	6.49E-04
4	30	F	44531	47268	-3	LSC;LSC	6.49E-04
5	30	F	87246	87269	-3	IRb;IRb	6.49E-04
6	30	P	87246	153134	-3	IRb;IRa	6.49E-04
7	30	P	87269	153157	-3	IRb;IRa	6.49E-04
8	30	F	89754	89775	-3	IRb;IRb	6.49E-04
9	30	P	89754	150628	-3	IRb;IRa	6.49E-04
10	30	P	89775	150649	-3	IRb;IRa	6.49E-04
11	30	F	150630	150651	-3	IRa;IRa	6.49E-04
12	30	F	153134	153157	-3	IRa;IRa	6.49E-04
13	31	P	64694	64737	0	LSC;LSC	1.48E-09
14	31	F	8329	35812	-2	LSC;LSC	6.20E-06
15	31	P	8329	45332	-2	LSC;LSC	6.20E-06
16	31	F	10539	36768	-3	LSC;LSC	1.80E-04
17	31	P	32327	32342	-3	LSC;LSC	1.80E-04
18	32	P	47813	47813	0	LSC;LSC	3.70E-10
19	33	F	92155	92203	-2	IRb;IRb	4.40E-07
20	33	P	92155	148197	-2	IRb;IRa	4.40E-07
21	33	P	92203	148245	-2	IRb;IRa	4.40E-07
22	33	F	148197	148245	-2	IRa;IRa	4.40E-07
23	33	P	76275	96435	-3	LSC;IRb	1.36E-05
24	33	F	76275	143965	-3	LSC;IRa	1.36E-05
25	33	P	124788	124788	-3	SSC;SSC	1.36E-05
26	34	R	126323	126323	-2	SSC;SSC	1.17E-07
27	39	F	43862	99493	-2	LSC;IRb	1.51E-10
28	39	P	43862	140901	-2	LSC;IRa	1.51E-10
29	41	F	38948	41172	-2	LSC;LSC	1.04E-11
30	52	P	30270	30270	0	LSC;LSC	3.37E-22
31	53	F	92159	92183	-1	IRb;IRb	1.34E-20
32	53	P	92159	148197	-1	IRb;IRa	1.34E-20
33	53	P	92183	148221	-1	IRb;IRa	1.34E-20
34	53	F	148197	148221	-1	IRa;IRa	1.34E-20
35	67	F	38916	41140	-3	LSC;LSC	4.06E-25
36	71	F	38908	41132	-3	LSC;LSC	1.89E-27
37	1626	P	84626	154181	0	IRb;IRa	0.00E+00
38	24665	P	86287	129481	0	IRb;IRa	0.00E+00
39	24699	P	86253	129481	-1	IRb;IRa	0.00E+00

Table S13 Long repetitive sequences in *P. zanlanscianense.*

Number	Length (bp)	Repeat type	Repeat start 1	Repeat start 2	Hamming	Region	E-value
1	30	P	9486	9486	-2	LSC;LSC	2.31E-05
2	30	F	89545	89608	-2	IRb;IRb	2.31E-05
3	30	P	89545	150442	-2	IRb;IRa	2.31E-05
4	30	P	89608	150505	-2	IRb;IRa	2.31E-05
5	30	F	150444	150507	-2	IRa;IRa	2.31E-05
6	30	F	9112	9152	-3	LSC;LSC	6.48E-04
7	30	F	43684	99391	-3	LSC;IRb	6.48E-04
8	30	P	43684	140659	-3	LSC;IRa	6.48E-04
9	30	F	87037	87060	-3	IRb;IRb	6.48E-04
10	30	P	87037	152990	-3	IRb;IRa	6.48E-04
11	30	P	87060	153013	-3	IRb;IRa	6.48E-04
12	30	F	152990	153013	-3	IRa;IRa	6.48E-04
13	31	P	64432	64475	0	LSC;LSC	1.48E-09
14	31	P	8324	45141	-1	LSC;LSC	1.37E-07
15	31	F	92045	92063	-1	IRb;IRb	1.37E-07
16	31	P	92045	147986	-1	IRb;IRa	1.37E-07
17	31	P	92063	148004	-1	IRb;IRa	1.37E-07
18	31	F	147986	148004	-1	IRa;IRa	1.37E-07
19	31	F	8324	35562	-2	LSC;LSC	6.18E-06
20	31	R	46780	46780	-2	LSC;LSC	6.18E-06
21	31	F	10539	36519	-3	LSC;LSC	1.79E-04
22	31	P	32086	32101	-3	LSC;LSC	1.79E-04
23	32	P	47640	47640	0	LSC;LSC	3.69E-10
24	32	R	46783	46786	-1	LSC;LSC	3.55E-08
25	32	P	35561	45141	-3	LSC;LSC	4.95E-05
26	33	F	89561	89603	-1	IRb;IRb	9.14E-09
27	33	P	89561	150444	-1	IRb;IRa	9.14E-09
28	33	P	89603	150486	-1	IRb;IRa	9.14E-09
29	33	F	89582	89603	-2	IRb;IRb	4.39E-07
30	33	P	89582	150444	-2	IRb;IRa	4.39E-07
31	33	P	89603	150465	-2	IRb;IRa	4.39E-07
32	33	F	91988	92036	-2	IRb;IRb	4.39E-07
33	33	P	91988	148011	-2	IRb;IRa	4.39E-07
34	33	P	92036	148059	-2	IRb;IRa	4.39E-07
35	33	F	148011	148059	-2	IRa;IRa	4.39E-07
36	33	P	76057	96321	-3	LSC;IRb	1.36E-05
37	33	F	76057	143726	-3	LSC;IRa	1.36E-05
38	33	P	124547	124547	-3	SSC;SSC	1.36E-05
39	34	R	126085	126085	-2	SSC;SSC	1.17E-07
40	36	F	150478	150499	0	IRa;IRa	1.44E-12
41	39	F	43672	99379	-2	LSC;IRb	1.50E-10

42	39	P	43672	140662	-2	LSC;IRa	1.50E-10
43	39	P	125849	125849	-3	SSC;SSC	5.56E-09
44	40	F	9120	9140	0	LSC;LSC	5.64E-15
45	41	F	38763	40987	-2	LSC;LSC	1.04E-11
46	49	F	89545	89587	-2	IRb;IRb	2.28E-16
47	49	P	89545	150444	-2	IRb;IRa	2.28E-16
48	49	P	89587	150486	-2	IRb;IRa	2.28E-16
49	49	F	150444	150486	-2	IRa;IRa	2.28E-16
50	52	P	30028	30028	0	LSC;LSC	3.36E-22
51	53	F	91992	92016	-1	IRb;IRb	1.34E-20
52	53	P	91992	148011	-1	IRb;IRa	1.34E-20
53	53	P	92016	148035	-1	IRb;IRa	1.34E-20
54	53	F	148011	148035	-1	IRa;IRa	1.34E-20
55	67	F	38731	40955	-3	LSC;LSC	4.05E-25
56	70	F	89545	89566	-3	IRb;IRb	7.23E-27
57	70	P	89545	150444	-3	IRb;IRa	7.23E-27
58	70	P	89566	150465	-3	IRb;IRa	7.23E-27
59	70	F	150444	150465	-3	IRa;IRa	7.23E-27
60	71	F	38723	40947	-3	LSC;LSC	1.89E-27
61	1112	P	84417	154551	0	IRb;IRa	0.00E+00
62	25302	P	85530	129248	0	IRb;IRa	0.00E+00

Table S14 RSCU values among six medicinal plants of *Polygonatum*.

Amino Acid	Codon	*P. zanlanscianense*	*P. kingianum*	*P. sibiricum*	*P. cyrtonema*	*P. filipes*	*P. odoratum*
L	CTA	0.831	0.818	0.837	0.84	0.841	0.841
	CTC	0.429	0.393	0.433	0.429	0.418	0.43
	CTG	0.359	0.351	0.366	0.364	0.362	0.361
	CTT	1.276	1.259	1.264	1.269	1.268	1.267
	TTA	1.86	1.943	1.845	1.844	1.846	1.848
	TTG	1.244	1.236	1.255	1.253	1.264	1.253
I	ATA	0.951	0.962	0.944	0.944	0.947	0.946
	ATC	0.62	0.593	0.618	0.619	0.618	0.619
	ATT	1.429	1.445	1.438	1.437	1.435	1.434
S	AGC	0.315	0.274	0.312	0.315	0.307	0.313
	AGT	1.199	1.298	1.201	1.2	1.204	1.201
	TCA	1.228	1.18	1.238	1.238	1.242	1.245
	TCC	1.018	1.004	1.002	1.016	1.013	1.011
	TCG	0.56	0.522	0.557	0.558	0.553	0.552
	TCT	1.68	1.721	1.69	1.673	1.681	1.678
G	GGA	1.624	1.596	1.633	1.632	1.636	1.634
	GGC	0.384	0.397	0.383	0.385	0.393	0.394
	GGG	0.681	0.661	0.673	0.669	0.662	0.667
	GGT	1.311	1.346	1.311	1.314	1.308	1.305
R	AGA	1.945	1.961	1.942	1.921	1.927	1.926
	AGG	0.593	0.587	0.598	0.606	0.603	0.599
	CGA	1.326	1.303	1.337	1.349	1.343	1.346
	CGC	0.313	0.314	0.303	0.307	0.314	0.318
	CGG	0.479	0.44	0.469	0.469	0.466	0.466
	CGT	1.344	1.395	1.351	1.349	1.346	1.346
F	TTC	0.768	0.753	0.772	0.769	0.774	0.773
	TTT	1.232	1.247	1.228	1.231	1.226	1.227
V	GTA	1.485	1.501	1.494	1.495	1.492	1.489
	GTC	0.492	0.478	0.487	0.485	0.482	0.482
	GTG	0.549	0.542	0.551	0.553	0.556	0.561
	GTT	1.474	1.479	1.468	1.467	1.47	1.467
A	GCA	1.187	1.173	1.176	1.164	1.174	1.168
	GCC	0.619	0.635	0.62	0.61	0.613	0.621
	GCG	0.403	0.401	0.414	0.414	0.416	0.414
	GCT	1.791	1.79	1.79	1.811	1.798	1.797
E	GAA	1.483	1.507	1.485	1.48	1.484	1.49
	GAG	0.517	0.493	0.515	0.52	0.516	0.51
K	AAA	1.481	1.5	1.486	1.486	1.486	1.487
	AAG	0.519	0.5	0.514	0.514	0.514	0.513

Continued Table

T	ACA	1.22	1.255	1.218	1.219	1.215	1.218
	ACC	0.734	0.729	0.731	0.733	0.729	0.735
	ACG	0.453	0.404	0.447	0.446	0.444	0.446
	ACT	1.594	1.612	1.604	1.602	1.611	1.601
N	AAC	0.484	0.479	0.485	0.488	0.488	0.489
	AAT	1.516	1.521	1.515	1.512	1.512	1.511
P	CCA	1.171	1.174	1.175	1.153	1.16	1.164
	CCC	0.84	0.865	0.851	0.858	0.847	0.852
	CCG	0.501	0.467	0.494	0.508	0.508	0.507
	CCT	1.488	1.494	1.481	1.481	1.484	1.477
D	GAC	0.41	0.417	0.41	0.409	0.405	0.407
	GAT	1.59	1.583	1.59	1.591	1.595	1.593
Y	TAC	0.413	0.422	0.415	0.411	0.413	0.412
	TAT	1.587	1.578	1.585	1.589	1.587	1.588
Q	CAA	1.511	1.507	1.508	1.506	1.505	1.504
	CAG	0.489	0.493	0.492	0.494	0.495	0.496
H	CAC	0.47	0.443	0.469	0.465	0.465	0.469
	CAT	1.53	1.557	1.531	1.535	1.535	1.531
M	ATG	1	1	1	1	1	1
W	TGG	1	1	1	1	1	1
C	TGC	0.443	0.453	0.45	0.448	0.448	0.448
	TGT	1.557	1.547	1.55	1.552	1.552	1.552

Table S15 Genbank ID of 59 species shown in the article.

Number	Species	Accession number	Length (bp)
1	*Polygonatum cyrtonema*	OL436258	155,205
2	*Polygonatum cyrtonema*	MW248135	155,816
3	*Polygonatum cyrtonema*	MZ029094	155,512
4	*Polygonatum cyrtonema*	MZ579646	155,596
5	*Polygonatum cyrtonema*	MZ150839	155,614
6	*Polygonatum cyrtonema*	This study	155,618
7	*Polygonatum franchetii*	OP615253	155,222
8	*Polygonatum macropodum*	NC_058562	154,610
9	*Polygonatum macropodum*	MZ150854	154,610
10	*Polygonatum odoratum*	MW248130	154,569
11	*Polygonatum odoratum*	OL405016	154,576
12	*Polygonatum odoratum*	MW248133	154,576
13	*Polygonatum odoratum*	This study	154,578
14	*Polygonatum involucratum*	OL405015	155,372
15	*Polygonatum involucratum*	NC_058560	155,370
16	*Polygonatum humile*	OL405013	155,185
17	*Polygonatum acuminatifolium*	NC_058569	155,354
18	*Polygonatum acuminatifolium*	MZ150867	155,354
19	*Polygonatum inflatum*	NC_058559	154,898
20	*Polygonatum inflatum*	MZ150848	154,898
21	*Polygonatum nodosum*	NC_058564	155,205
22	*Polygonatum nodosum*	MZ150857	155,205
23	*Polygonatum filipes*	MZ150842	155,317
24	*Polygonatum filipes*	MZ150843	155,337
25	*Polygonatum filipes*	MZ571521	155,336
26	*Polygonatum filipes*	ON534062	155,361
27	*Polygonatum filipes*	This study	155,333
28	*Polygonatum sibiricum*	MZ029093	155,512
29	*Polygonatum sibiricum*	MW373521	155,549
30	*Polygonatum sibiricum*	ON534064	155,514
31	*Polygonatum sibiricum*	OQ532972	155,513
32	*Polygonatum sibiricum*	OL405024	155,514
33	*Polygonatum sibiricum*	This study	155,572
34	*Polygonatum kingianum*	OQ532971	155,793
35	*Polygonatum kingianum*	MW566464	155,796
36	*Polygonatum kingianum*	MW566456	155,738
37	*Polygonatum kingianum*	MW373517	155,824
38	*Polygonatum kingianum*	OQ532967	155,802
39	*Polygonatum kingianum*	MZ029091	155,802
40	*Polygonatum kingianum*	This study	155,807
41	*Polygonatum stenophyllum*	KX822773	156,028
42	*Polygonatum stenophyllum*	OL405025	155,961
43	*Polygonatum tessellatum*	NC_058567	155,688
44	*Polygonatum punctatum*	NC_058566	155,657
45	*Polygonatum punctatum*	OQ532974	155,437
46	*Polygonatum zanlanscianense*	ON534059	155,787
47	*Polygonatum zanlanscianense*	OL405020	155,827

Continued Table

48	*Polygonatum zanlanscianense*	MW373522	155,911
49	*Polygonatum zanlanscianense*	This study	155,663
50	*Polygonatum hookeri*	MZ150846	155,956
51	*Polygonatum hookeri*	OL405012	155,953
52	*Polygonatum cirrhifolium*	MN912821	156,002
53	*Polygonatum verticillatum*	MZ150866	155,856
54	*Polygonatum prattii*	OQ532969	155,839
55	*Polygonatum prattii*	OL405017	155,887
56	*Polygonatum curvistylum*	OQ532970	155,891
57	*Polygonatum curvistylum*	MZ150837	155,939
58	*Dioscorea aspersa*	NC_039807	153,337
59	*Dioscorea alata*	NC_039707	153,161
